# Collection of Aerosolized Human Cytokines Using Teflon® Filters

**DOI:** 10.1371/journal.pone.0035814

**Published:** 2012-05-04

**Authors:** Jennifer H. McKenzie, James J. McDevitt, M. Patricia Fabian, Grace M. Hwang, Donald K. Milton

**Affiliations:** 1 Biomedical Engineering and Biotechnology Program, University of Massachusetts, Lowell, Massachusetts, United States of America; 2 Department of Environmental Health, Harvard School of Public Health, Boston, Massachusetts, United States of America; 3 The MITRE Corporation, McLean, Virginia, United States of America; 4 Maryland Institute for Applied Environmental Health, School of Public Health, University of Maryland, College Park, Maryland, United States of America; French National Centre for Scientific Research, France

## Abstract

**Background:**

Collection of exhaled breath samples for the analysis of inflammatory biomarkers is an important area of research aimed at improving our ability to diagnose, treat and understand the mechanisms of chronic pulmonary disease. Current collection methods based on condensation of water vapor from exhaled breath yield biomarker levels at or near the detection limits of immunoassays contributing to problems with reproducibility and validity of biomarker measurements. In this study, we compare the collection efficiency of two aerosol-to-liquid sampling devices to a filter-based collection method for recovery of dilute laboratory generated aerosols of human cytokines so as to identify potential alternatives to exhaled breath condensate collection.

**Methodology/Principal Findings:**

Two aerosol-to-liquid sampling devices, the SKC® Biosampler and Omni 3000™, as well as Teflon® filters were used to collect aerosols of human cytokines generated using a HEART nebulizer and single-pass aerosol chamber setup in order to compare the collection efficiencies of these sampling methods. Additionally, methods for the use of Teflon® filters to collect and measure cytokines recovered from aerosols were developed and evaluated through use of a high-sensitivity multiplex immunoassay. Our results show successful collection of cytokines from pg/m^3^ aerosol concentrations using Teflon® filters and measurement of cytokine levels in the sub-picogram/mL concentration range using a multiplex immunoassay with sampling times less than 30 minutes. Significant degradation of cytokines was observed due to storage of cytokines in concentrated filter extract solutions as compared to storage of dry filters.

**Conclusions:**

Use of filter collection methods resulted in significantly higher efficiency of collection than the two aerosol-to-liquid samplers evaluated in our study. The results of this study provide the foundation for a potential new technique to evaluate biomarkers of inflammation in exhaled breath samples.

## Introduction

Chronic respiratory diseases such as asthma, chronic obstructive pulmonary disease (COPD), and lung cancer affect hundreds of millions of people worldwide and cause over four million deaths annually [1]. Although many risk factors have been identified for both asthma and COPD, a complete understanding of the underlying disease mechanisms has not yet been achieved for either chronic disease. Additionally, improvements in tools used for diagnosing and managing these diseases are urgently needed [2], [3], [4].

Exhaled breath is an aerosol consisting mostly of water vapor, with smaller amounts of volatile, semi-volatile, and non-volatile molecules derived from the upper and lower portions of the respiratory system [5], [6]. Cytokines are small, water-soluble signaling proteins produced by cells of the immune system to modulate responses of the immune system such as inflammation. Since inflammation is an underlying condition of many chronic diseases, exhaled cytokines can be considered biomarkers of pulmonary inflammation that could indicate the presence of lung diseases or provide information regarding the current status of the lungs. Non-invasive monitoring of lung inflammation through detection and measurement of cytokines in exhaled breath samples is a promising new approach aimed at addressing the need for improved understanding, treatment and management of chronic respiratory diseases such as asthma and COPD.

Over the last 15 years, there has been increasing interest in the development and use of exhaled breath condensate (EBC) techniques for the collection and analysis of aerosolized droplets of respiratory lining fluid (RLF). Collection of EBC samples is a non-invasive and relatively well-tolerated procedure accomplished through simple means whereby a subject breathes normally into a chilled collection device that condenses and collects the fluid samples. EBC samples consist of a mixture of three main components [6]. The most abundant component (99%) of EBC samples is liquid water formed from the condensation of water vapor present in the warm exhaled air, saturated with water vapor as it leaves the respiratory tract. The second and third components of EBC samples are water-soluble volatiles and non-volatile particles that are aerosolized from the respiratory lining fluid and are present in significantly smaller amounts than the water component of EBC samples [6], [7], [8], [9], [10], [11]. The significant amount of liquid water present in EBC samples dilutes the inherently low concentrations of non-volatile biomarkers to levels that are at or below the detection threshold of most commercially available assays [6]. The inefficient collection of exhaled, nonvolatile submicron particles using EBC methods combined with assay sensitivity limitations creates significant problems with reproducibility and validity of biomarker measurements [6], [10], [11], [12], [13], [14]. Several recent papers suggest that collection of respiratory particles by more efficient methods may be feasible and avoid some of the problems with EBC including variable collection efficiency with variable tidal flow [15], [16], [17], [18].

Since exhaled breath is a bioaerosol and liquid-based collection methods may preserve biological function of protein biomarkers, aerosol-to-liquid based sampling devices might be useful for collection of exhaled breath samples. Liquid-impinger sampling devices collect aerosolized particles into a liquid medium through inertial impaction. The use of liquid collection medium prevents desiccation and possible degradation of collected particles, however, the forces used for liquid-impingement collection can be destructive to biological molecules [19]. One well-known and frequently used liquid-impingement aerosol sampler, the BioSampler® (SKC Inc., Eighty Four, PA), utilizes centrifugal forces for more gentle collection of particles and is widely recognized for its ability to preserve biological function. However, key disadvantages associated with the BioSampler® include its relatively low sample flow rate and high sample collection volume which results in considerable dilution of collected particles. Another technology offering the benefits of liquid-based sample collection is the wetted-wall cyclone. In this method, aerosolized particles are subjected to centrifugal forces whereby particles are deposited onto the wetted wall of the sampling device due to inertial forces. Since this device utilizes a relatively high sample flow rate combined with a relatively low sample collection volume, it may offer improved collection over liquid-impingement collection devices. However, the suitability of this collection method to aerosolized non-volatile particles such as cytokines has not yet been established or compared to liquid-impingement aerosol collectors.

Filters collect particles through a variety of mechanisms. Direct interception and impaction favor larger particles, while diffusion and electrostatic forces favor small submicron particles. Thus, filters are capable of collecting a wide range of particle sizes. Impingers and cyclones favor collection of large particles and typically have low collection efficiencies for submicron particles [19], [20]. Given that a number of recent studies have consistently demonstrated that the majority of exhaled breath particles are in the submicron (<1 µm) range [21], [22], [23], the use of filters for collection of exhaled breath samples may offer a promising new efficient means for collection of non-volatile cytokines. Recent work has successfully demonstrated the ability to collect influenza particles from aerosols generated with a nebulizer and one-pass aerosol chamber [24] and from exhaled human breath samples using Teflon® filters [17]. Additionally, successful collection and analysis of exhaled breath biomarkers has been demonstrated using silicon plates and Time-of-Flight Secondary Ion Mass Spectrometry (TOF-SIMS) [25] and ELISA [15], [16]. These studies suggested that collection and measurement of human cytokine aerosols generated in a similar manner might be possible using filter-based methods and may be much more efficient at recovery of respiratory fluid biomarkers than condensate [15].

In this paper, we report the sampling and detection of human cytokine aerosol generated using a nebulizer and transported through a single-pass aerosol chamber. A high volume chamber flow was used to dilute the cytokine aerosol to more realistic levels. We examine the collection efficiency for human cytokine aerosols of Teflon® filters and two aerosol-to-liquid samplers: the Biosampler® and the Omni 3000™. We also report results of sampling a range of aerosolized cytokine concentrations for the purpose of generating filter samples containing cytokine levels below, near and above the detection limits of a high-sensitivity multiplex immunoassay. With this data, we assess the variability of collection and assay methods at low cytokine levels likely to be encountered in human exhaled breath studies. Finally we evaluate the effect of storage conditions, such as would be required by most clinical or epidemiologic field studies, on degradation of cytokines collected with Teflon® filters.

Methods developed and optimized as a result of this study may be used to evaluate the feasibility of filter-based collection methods to collect and measure human cytokines in exhaled breath samples and offer a novel method for monitoring pulmonary biomarkers of inflammation.

## Results

### Experiment 1: Estimation of Aerosolized Cytokine Concentrations and Comparison of Aerosol Sampler Collection Efficiency

**Figure 1:**
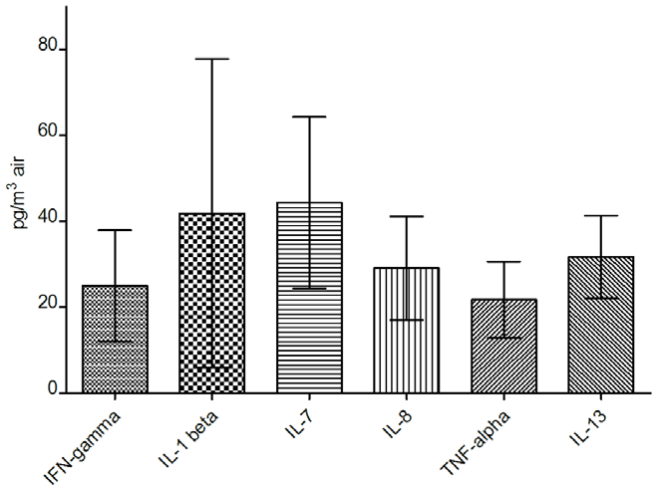
Estimated airborne concentrations of six human cytokines produced in single-pass aerosol chamber. Data shown for estimated expected airborne concentrations (pg/m^3^ air) of six different human cytokines as calculated by Equation 1 (N = 2; 95% CI).

Estimated aerosol concentrations in the single pass aerosol chamber for all cytokines, based on measurements of cytokines in the nebulizer before and after each run, were typically in the 20–40 pg/m^3^ range with notable cytokine-dependent variability (Figure 1).

**Figure 2:**
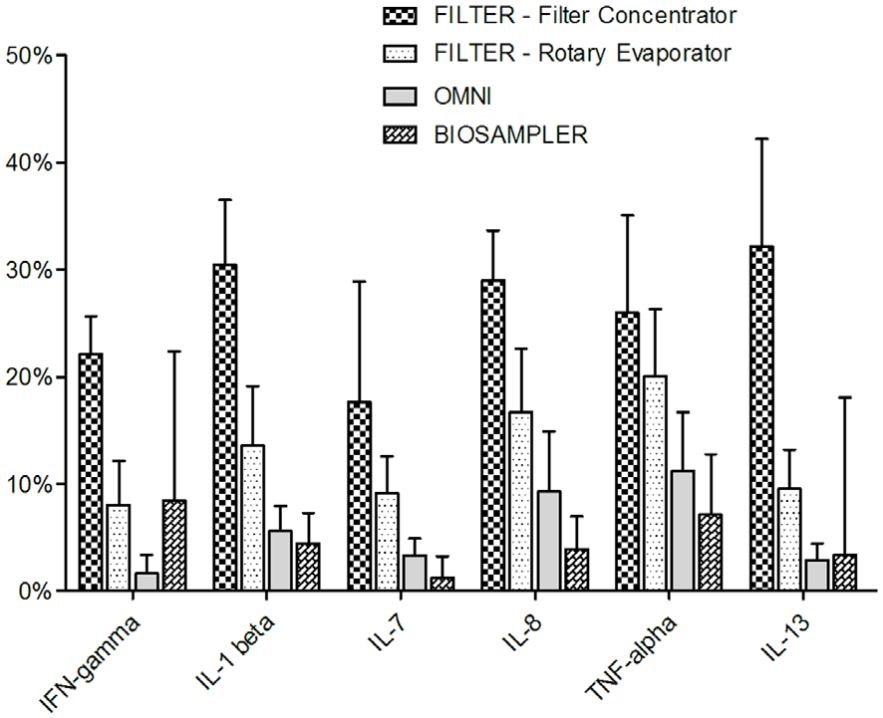
Comparison of sampling device collection efficiencies by cytokine. Collection efficiencies calculated using Equation 3 for each of six different human cytokines using different collection devices in a single-pass aerosol chamber (N = 6; 3 filters assayed in duplicate, 95% CI).

**Figure 3:**
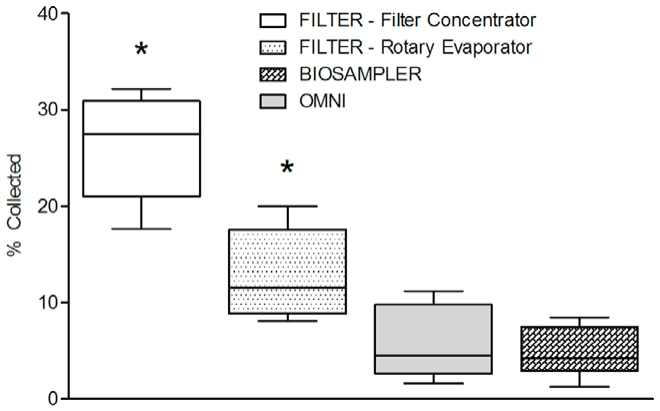
Comparison of mean collection efficiencies by sampling device type. Tukey Box Plot of mean collection efficiencies calculated using Equation 3 of six human cytokines using different collection devices in a single-pass aerosol chamber (N = 36; 3 filters assayed in duplicate for each of 6 different cytokines, 95% CI). * indicates significance at P<0.05.

All measured cytokine levels in Experiment 1 were above the lowest standard of the assay (0.03 pg/mL). Variability of cytokine level measurements was high for all cytokines and sampling devices (Figure 2). Overall collection efficiency was poor (<20%) for all cytokines using all three methods: the Biosampler®, the Omni-3000™ and filters concentrated using rotary centrifugal evaporation (Figures 2, 3). For all cytokines tested, filter collection yielded higher collection efficiencies than either the Omni 3000™ or SKC Biosampler® (Figures 2, 3). IL-7 seemed to be particularly difficult to recover using the SKC Biosampler® while IFN-gamma was difficult to recover using the Omni-3000 (Figure 2). Significant differences in mean collection efficiencies of all six cytokines among the three samplers were observed (Figure 3, Kruskal-Wallis ANOVA, P<0.05). Subsequent paired student’s t-tests indicated significantly improved mean collection efficiency using filters as compared with mean collection efficiencies of either the Omni™-3000 (P<0.0001) or Biosampler® (P = 0.01), Figure 3). No significant difference in mean collection efficiency was observed between the Omni 3000™ and Biosampler® (P = 0.64, Figure 3). The coefficient of variation (CV) calculated for cytokine measurements on samples collected from filters and concentrated using rotary evaporation, Biosampler® and Omni 3000™ were 37%, 55% and 68%, respectively.

### Experiment 2: Cytokine Stability, Use of Filter Centrifugation Concentration and Effect of Sampling Time and Cytokine Concentrations on Assay Repeatability

Degradation of cytokines in solution at 25 pg/mL was found to be insignificant over 30 minutes of nebulization at 68.9 kPa (Kruskal-Wallis ANOVA, P>0.05, Figure S1).

Spike-recovery experiments evaluating the use of Amicon Ultra-4 3000 MWCO centrifugal filter concentrators indicated approximately 80% sample recovery as compared to approximately 50% recovery of sample using rotary centrifugal evaporation methods. Passivation of centrifugal filter concentrators did not result in significantly higher recovery of cytokines as compared to untreated filters (student’s t-test, P>0.05).

Filter samples collected from aerosolization of cytokines at 25 pg/mL and concentrated using centrifugal filter concentrators displayed significantly improved collection efficiency as compared to filter samples collected and concentrated using rotary centrifugal evaporation (Figure 3, t-test, P = 0.003). Mean collection efficiency of filter extracts concentrated using centrifugal filter concentrators was approximately twice that of filter extracts concentrated using rotary evaporation (Figure 3, 26% versus 13%) and nearly 5-fold higher than the collection efficiency of the Omni-3000™ (5.6%) and the Biosampler® (4.8%). As in Experiment 1, cytokine-specific differences in collection efficiency were observed when using the centrifugal filter concentrators (Figure 2). Additionally, the CV of filter sample extracts concentrated using centrifugal filter concentrators was lower than that of extracts concentrated with rotary evaporation (21% versus 37%).

**Figure 4:**
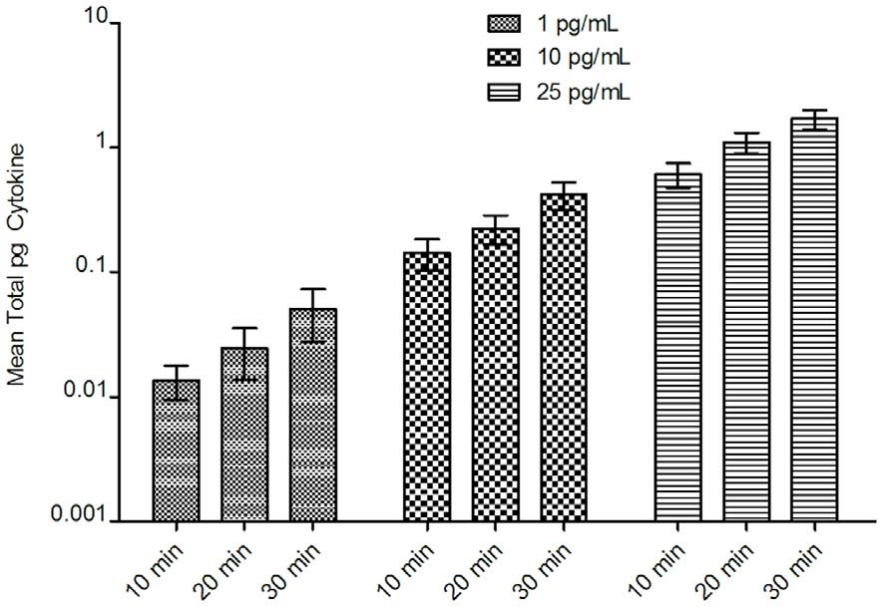
Effect of sampling time and initial starting concentration on cytokine collection. Mean cytokine levels (pg) of six human cytokines collected using Teflon filters in aerosol chamber for 10, 20 and 30 minutes at starting concentrations of 1, 10 and 25 pg/mL. 95% CI bars are shown (N = 48, 4 filters assayed in duplicate for each of 6 cytokines).

Filter samples were collected from aerosolized human cytokines that yielded measured cytokine levels below, near 10-fold and 100-fold above the assay manufacturer’s reported MinDC of the high-sensitivity multiplex immunoassay (Figure 4). All cytokine levels measured from filters collected from aerosolization of cytokines at 25 pg/mL were above the lowest standard of the assay (0.03 pg/mL; observed range 0.7 pg/mL to 39.7 pg/mL). Three sample cytokine level measurements (2%) from filters taken from aerosolization of cytokines at 10 pg/mL (observed range 0.2 pg/mL to 13 pg/mL) and 35 cytokine level measurements (26%) from filters taken from aerosolization of cytokines at 1 pg/mL displayed levels below the lowest standard of the assay (0.03 pg/mL; observed range 0.03 pg/mL to 4.4 pg/mL). Significant increases in cytokine collection corresponded with increased starting concentration and increased sampling time (Figure 4, Kruskal-Wallis ANOVA, P<0.05). Additionally, repeatability of measurements increased with increasing cytokine concentrations as indicated by 95% CI bars (Figure 4).

Significant differences in measured cytokine levels were observed among the six cytokines tested in the study. (Figures S2, S3, S4, S5, S6, S7, Kruskal-Wallis ANOVA, P<0.05). However, the trends were the same for all cytokines tested. Each cytokine displayed significant increases in collected amount at increased starting concentrations and sampling times (Figures S2, S3, S4, S5, S6, S7, Kruskal-Wallis ANOVA, P<0.05).

Repeatability of measurements, measured as a reduction in CV, was improved by increasing starting concentrations. However, longer sampling times did not appear to significantly improve repeatability of measurements.

Blank filter samples that received no air exposure contained no detectable levels of cytokines.

### Experiment 3: Effect of Storage Conditions

**Figure 5:**
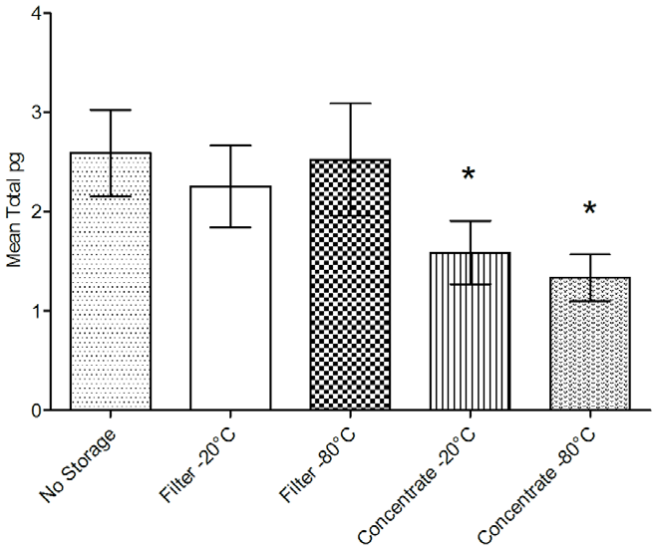
Effect of storage condition on mean cytokine levels. Mean cytokine levels (pg) across five different storage conditions (95% CI, N = 156, 6 filters assayed in duplicate for each of 13 human cytokines). * indicates statistically significant difference from No Storage control condition (Kruskal-Wallis ANOVA, P<0.05).

All measured cytokine levels were above the lowest standard of the assay (0.03 pg/mL). Mean cytokine levels of thirteen different cytokines varied significantly with storage condition (Figure 5, Kruskal-Wallis ANOVA, P<0.001). Comparisons made using student’s t-tests of individual pairs of storage conditions showed no significant difference between samples receiving no storage (control condition) and filter samples stored at either −20°C or −80°C. However, significant decreases in cytokine levels were observed for concentrated samples stored at either −20°C or −80°C when compared to samples receiving no storage (P≤0.0005) or when compared to filter samples stored at −20°C (P<0.05) or filter samples stored at −80°C (P<0.01). No significant difference was observed between concentrate samples stored at −20°C or −80°C (P = 0.20).

Individual analyses of thirteen cytokines revealed similar responses to storage conditions (Figures S8, S9, S10, S11, S12, S13, S14, S15, S16, S17, S18, S19, S20), with increased degradation of cytokines observed in concentrate samples stored at both −20°C and −80°C. Total amounts of GM-CSF, IFN-gamma, IL-1 beta, IL-2, IL-4, IL-5, IL-7, IL-8, varied significantly with storage condition (Kruskal-Wallis ANOVA, P<0.05). Total amounts of IL-6 displayed borderline significant differences across storage conditions (Kruskal-Wallis ANOVA, P = 0.06).

Blank filter samples that received no air exposure contained no detectable levels of cytokines.

## Discussion

The diagnosis and treatment of pulmonary diseases would benefit greatly from a sensitive, non-invasive method of identification and measurement of biomarkers of pulmonary inflammation creating much interest in the collection of exhaled breath particles for measurement of cytokines. A number of recent studies have shown a predominance of exhaled breath particles in the submicron range [21], [22], [23]. Collection of exhaled breath particles through condensation using EBC techniques or through the use of aerosol-to-liquid based sampling devices such as the Biosampler® and Omni 3000™ are not very efficient collectors of submicron particles [20]. Based on aerosol theory, filters are expected to outperform these collections systems for particles in the submicron range and offer the potential for development of novel exhaled breath collection systems and methodologies.

Our results indicate that collection of aerosolized human cytokines is feasible using Teflon® filters at airborne concentrations in the pg/m^3^ air range. The ability of this filter-based method to capture and detect human cytokines at such low concentrations suggests the possibility of utilizing this technique for collection and measurement of cytokines in exhaled breath samples. The use of Teflon® filters improved the collection of human cytokine aerosols by approximately 5-fold as compared to traditional aerosol-to-liquid-samplers such as the Biosampler® and Omni-3000™ (Figures 2 and 3). Additionally, variability of cytokine measurements for both the Biosampler® and Omni-3000™ were much higher than that of filter-based collection when extracts were concentrated using centrifugal filter concentration (55% and 68%, respectively versus 21%). Differences in collection efficiency may be explained by decreased ability of the aerosol-to-liquid samplers to capture submicron particles. A recent review article cited a study that estimated liquid-based sampling devices lose as much as 90% of submicron particles [20]. However, individual collection efficiencies will vary based on sampler design and operating parameters. Increased variability observed in the liquid-sampling methods may also be due to decreased collection efficiencies that produced lowered measurable concentrations of cytokines that were closer to the sensitivity limits of the immunoassay. Increased efficiency and decreased variability of collection of non-volatile biomarkers through the use of filter-based collection methods could increase the amount of cytokine available for subsequent detection and quantitative assays. These improvements may help address the reproducibility and validity problems encountered in exhaled breath condensate methods due to measurement of biomarkers at concentrations near assay detection limits [12], [13], [14].

Our study suggests that cytokines can be detected from aerosols at levels near the MinDC of the high-sensitivity multiplex immunoassay (Figure 4, Figures S2, S3, S4, S5, S6, S7). This represents the potential to detect cytokines in the sub-picogram/mL range from aerosols containing cytokines in the picogram/m^3^ range with sampling times of 30 minutes or less (Figures 1 and 5, Figures S2, S3, S4, S5, S6, S7). Detection of cytokines at this level of sensitivity is of importance since the content of exhaled breath samples has been estimated to contain 99.99% water vapor with non-volatile molecules comprising <0.01% of exhaled breath condensate samples [8]. Higher variability of cytokine measurements made at the lower cytokine concentrations (Figure 4, Figures S2, S3, S4, S5, S6, S7) may not likely be useful for quantitative purposes, but may be within an acceptable range for detecting significant differences in cytokine concentrations among individuals or determining the presence or absence of various cytokines in various samples.

In this study we evaluated four different storage conditions that were selected based on the most likely workflow that would be implemented in sample processing protocols. Results of this study indicate that storage conditions should be evaluated for each cytokine in a study when developing a cytokine measurement assay method. Our study displayed significantly decreased levels of cytokine for those samples stored as concentrated filter extracts at −20°C or −80°C (Figure 5, Figures S8, S9, S10, S11, S12, S13, S14, S15, S16, S17, S18, S19, S20). Additionally, results of our study show that cytokines vary significantly in their susceptibility to degradation during storage (Figures S8, S9, S10, S11, S12, S13, S14, S15, S16, S17, S18, S19, S20). Other studies have also shown the importance of handling and storage of cytokine samples and demonstrated differences in susceptibility among cytokines [26], [27], [28]. Potential causes for the observed differences in stability were speculated to be cytokine tertiary or quaternary structural variations or components of the sample matrix [28]. Our results also suggest a significant decrease in recovered cytokine amounts when storing samples as concentrated filter extracts at either −20°C or −80°C versus filters stored at −20°C or −80°C or samples receiving no storage (Figure 5). However, due to variability in assay measurements, further study is warranted to confirm the validity of this finding. A recent study by Weist (2010) evaluating protein storage and handling conditions concluded that tissue samples could be stored at −80°C for years without significant degradation, but extracts frozen at −80°C and thawed showed significant degradation [29]. Our study concurs with the results of Weist’s study indicating significantly higher degradation of stored extracts as compared to filter samples stored at the same temperature. Potential causes for this decrease could be due to degradation of proteins in the presence of water and oxygen or loss of sample to the storage tube.

A limitation of our study was use of optimized, experimental conditions with an artificial aerosol diluted in a large air flow to simulate concentrations that might be encountered in exhaled breath. We did not match the temperature and humidity of exhaled breath in this system. The constant air flow in our sampling devices differed from cyclically variable flow in EBC devices, but was similar to that in more recent exhaled breath sampling devices used by our lab and others [15], [16], [17], [18]. We used BSA to mimic the concentration of proteins in respiratory lining fluids and used Tween-20 as a surfactant rather than trying to match the mixture of proteins and surfactant proteins and lipids present in the distal airways. Thus, these artificial conditions can only explore the feasibility of new collection methods as alternatives to currently popular EBC methods. Future studies to explore the utility of this method for collection of exhaled breath particles from human subjects will be required before the method can be more widely adopted for research.

Another limitation of our study was the small number of sampler types evaluated for the collection of non-volatile particles from bioaerosols. Other types of samplers exist and should be evaluated; e.g. Mainelis (2002) demonstrated efficient collection bioaerosols while preserving biological function through the use of an electrostatic precipitator collection device [30]. In contrast to bioaerosol impactors and impingers, electrostatic precipitators offer the ability to collect samples in a liquid medium at particle velocities 2–4 orders of magnitude lower than impactors and impingers and thus offer the ability to collect particles in a manner that may aid in preserving biological function. Additionally, Han and Mainelis (2010) have shown the ability to use an electrostatic precipitator with superhydrophobic collection surface (EPSS) to collect bioaerosol samples into concentrated volumes to allow for detection of molecules present at very low concentrations [31]. These studies provide another feasible approach for collection of aerosolized human cytokines at low concentrations while preserving biological activity that could be evaluated in future work. A newer technology recently reviewed by Adler (2008) combining immunoassay with nucleic acid amplification allows for detection of biological molecules at concentrations as low as 1 femtogram/mL offering a 100–1000X increase in sensitivity over current methods [32]. Application of this new technology to measurement of cytokines in exhaled breath samples may provide the assay sensitivity and reproducibility needed for cytokine profiling in chronic pulmonary disease and warrants further study.

### Conclusions

Our study demonstrated successful collection of aerosolized human cytokines in the pg/m^3^ concentration range using filter based methods and measurement of cytokine levels in the sub-picogram/mL range using a high-sensitivity multiplex immunoassay utilizing sampling times of 10 to 30 minutes. Use of Teflon® filters yielded significant improvements in collection of aerosolized human cytokines as compared to two standard aerosol-to-liquid- collection methods. This finding suggests a new method of exhaled particle collection that may collect significantly more particles than popular exhaled breath condensate collection methods. The improved collection efficiency gained through use of filters may enable collection of sufficient material to result in measurement of cytokines at levels above current assay detection limits, ultimately leading to more reliable and repeatable assay data.

Our results also highlight the need to evaluate handling and storage of cytokines to minimize degradation of samples when developing methods for cytokine measurement. We observed a potential increase in degradation of proteins in samples stored as extracts at either −20°C or −80°C, a finding that concurs with other recent work in this area. This may suggest that filter samples containing cytokines should not be stored as concentrated extracts, but as dry samples on filters at −20°C or −80°C until ready for assay. Additional studies to confirm the validity of our finding and are necessary to determine optimal storage of cytokine-containing samples. Our findings provide the foundation for a potential new technique to detect and measure cytokines and other non-volatile pulmonary inflammatory biomarkers in human exhaled breath samples.

**Figure 6:**
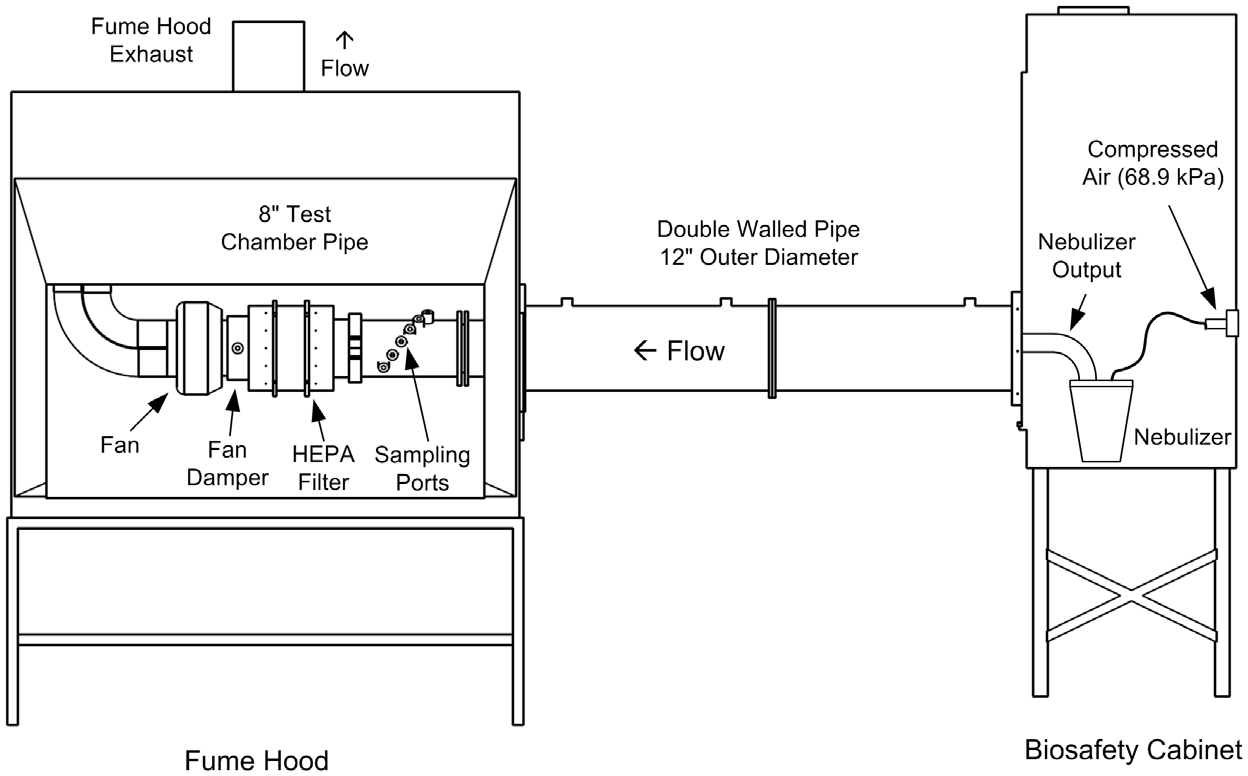
Schematic diagram of single-pass aerosol chamber with HEART nebulizer. Single-pass aerosol chamber set up used for nebulization and collection of aerosolized human cytokines. 2010©-The MITRE Corporation Approved for Public Release: 10–1260. Distribution Unlimited.

**Figure 7:**
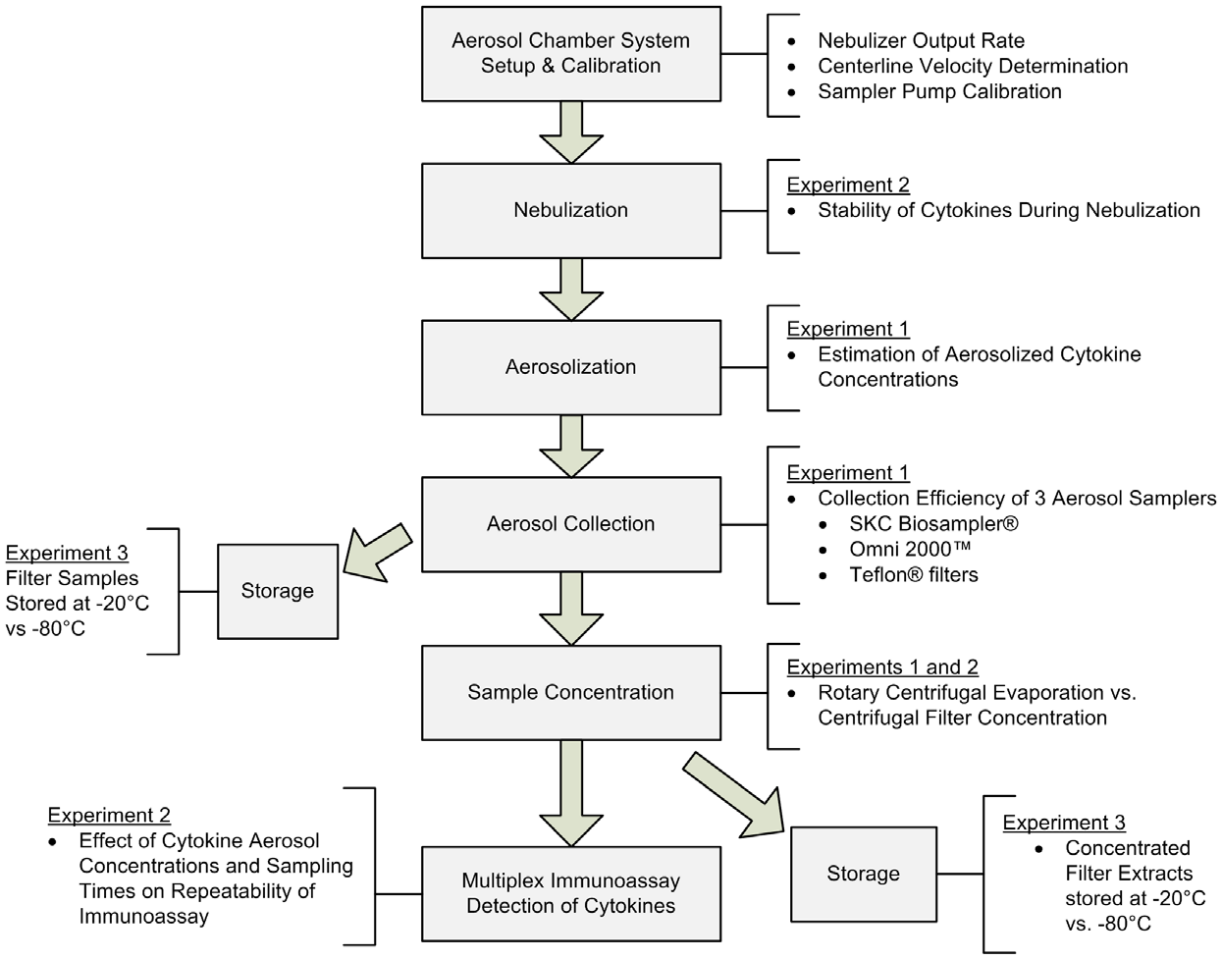
Summary of experiments and methods.

## Materials and Methods

### Overview

A single-pass aerosol chamber with biosafety hood for nebulization of cytokine solutions was set up as shown in Figure 6. Briefly, cytokine aerosols were generated with a HEART nebulizer and dried while traveling through approximately 70 inches (1.78 m) of duct until reaching the sampling ports. A high protein concentration in the nebulizer simulated respiratory lining fluid source of respiratory droplets. Sampling ports used in the chamber were designed to allow for isokinetic sampling of aerosols. Three separate experiments were conducted using this system evaluating a variety of factors influencing the collection, handling and storage of human cytokine aerosols and are summarized in Figure 7.

### Initial Setup, Calibration and Evaluation of Aerosol Generation and Collection System

A High-Output Extended Aerosol Respiratory Therapy (HEART, Westmed Inc., Tucson, AZ) nebulizer was used for nebulization of a mixed cytokine solution at 68.9 kPa based on previous work demonstrating successful aerosol generation at this pressure. The nebulizing solution contained 100 mL of 1X Phosphate-Buffered Saline (PBS, Sigma-Aldrich, St. Louis, MO), 1% Bovine Serum Albumin (BSA; Sigma-Aldrich, St. Louis, MO) and 0.01% (v/v) Tween-20 (Tween buffer; Polyethylene glycol sorbitan monolaurate; Sigma-Aldrich, St. Louis, MO. The output rate of the HEART nebulizer at 68.9 kPa was characterized for stability over 30 mins by weighing the solution at 0, 10, 20 and 30 minutes and calculating amount of solution removed from the nebulizer (Figure 7, Figure S21). The mean output rate of the HEART nebulizer over 30 minutes was 0.32 g/min (Figure S21, SD = 0.06 g/min).

The centerline velocity of the 8 in duct of the aerosol chamber was measured and recorded. The velocity profile for an 8 in duct of the aerosol chamber was performed taken from a traverse of 10-point measurements as described in the Pitot Traverse Method for Round Pipe (ACGIH, Industrial Ventilation 23rd Ed.) from which an average velocity was calculated (Figure 7). The centerline velocity associated with the average velocity of the chamber calculated from the pitot traverse was then used as a reference value for setting the chamber flow rate. Measured centerline velocity was 57 F/min (17.4 m/min). Calculated average velocity was 53.6 F/min (16.3 m/min) which differed 6% from measured centerline velocity.

Vacuum pumps used with each sampling device (Figure 7) were calibrated using a rotometer (Part Number FM044040, Cole-Parmer, Vernon Hills, IL) which was first calibrated by a soap flow-meter (Gilibrator, Part Number D800285, Gillian Instrument Corp., West Caldwell, NJ). Pumps were evaluated for flow stability for 30 minutes. Mean % change in vacuum pump flow rate was 2.4% (SD  =  1.1%, min  =  0.7%, max  =  3.9%).

### Multiplex Immunoassay Analysis

All samples were assayed using a high sensitivity human cytokine LINCOplex Kits (Millipore, Billerica, MA) according to the manufacturer’s instructions and analyzed using a Luminex 200 IS System (Luminex Corporation, Austin, TX). All samples and cytokine standards were assayed in a solution of 1X PBS/1% BSA/0.01% Tween-20. Cytokine concentrations (pg/mL) for all experiments were calculated using Upstate Beadview (Temecula, CA) software.

### Units of Measurement

Concentrations of prepared cytokine solutions are described in pg/mL. Airborne concentrations of cytokines are reported in pg/m^3^ air and provide a useful context for interpretation of sampler collection efficiencies and reported assay measurements. All other reported results are presented in total mass (pg) of cytokine measured or collected by the sampling device.

### Experiment 1: Estimation of Aerosolized Cytokine Concentrations and Comparison of Aerosol Sampler Collection Efficiency

A HEART nebulizer was employed to generate aerosols of six human cytokines (IFN-gamma, IL-1 beta, IL-7, IL-8, IL-13, and TNF-alpha; Millipore, St. Charles, MO) at 68.9 kPa from a 100 mL solution containing 50 pg/ml of each cytokine in 1X PBS buffer prepared as described previously. Samples were removed from the nebulizer solution using a syringe attached to a separate sampling port located on the top of the HEART nebulizer that did not interfere with nebulization.

Using Luminex assay data, concentrations of each cytokine present in the HEART nebulizer solution before and after nebulization were determined. Estimations of expected airborne cytokine concentrations (Figure 7) were then calculated as follows:



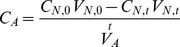
(1)Where *C_A_* is the expected airborne cytokine concentration (pg/m^3^), *C_N,0_* is the pre-nebulization cytokine concentration in the nebulizer (pg/mL) and *C_N,t_* is the post nebulization concentration (pg/mL), *V_N,0_* is the volume of fluid in the nebulizer pre-nebulization (mL) and *V_N,t_* is the volume post-nebulization (mL), and *V_A_*-dot is the average air flow in the chamber(m^3^/min) and t is the duration of the experiment (min).

Mean airborne cytokine concentrations and 95% CI values for each cytokine collected in the study were calculated.

From the same aerosol generated for the purpose of estimating cytokine aerosol concentrations, we tested the collection efficiency of the BioSampler® (SKC Inc., Eighty Four, PA); the Omni-3000™ (Sceptor Industries, Kansas City, MO); and Teflo™ 2.0 micron Teflon® filters (Pall Corporation, East Hills, NY) housed in 37 mm polypropylene cassettes using the same chamber set up (Figures 6 and 7). Samples were concurrently collected for 15 minutes. Tunnel flow rate was 566 L/min and sampler flow rates for the BioSampler®, Omni-3000 and filter cassettes were 12.5 L/min, 252 L/min, and 28.3 L/min, respectively.

Aliquots of 1 mL were taken directly from the BioSampler® and Omni-3000™ using a syringe. Filter washes were performed immediately after collection as follows. First, filters were cut with scissors around perimeter by making a series of notches in the polypropylene support ring. Next, each filter was placed in 1.5 mL polypropylene centrifuge tubes and rinsed by vortexing in 1 mL of 1X PBS/1% BSA/0.01% Tween-20 solution. All samples were then concentrated 10-fold using a centrifugal rotary evaporator.

Cytokine concentrations were normalized for air volume, liquid collection volume, and filter extraction volume. The measured concentration of cytokine collected by each sampling device was then calculated as described below in Equation 2.

Equation 2.
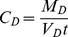
(2)Where *C_D_* is the measured concentration of cytokine collected by the sampling device, *M_D_* is the average total mass of cytokine collected by the sampling device based on reported immunoassay concentrations, *V_D_*-dot is the air flow to the sampling device, and *t* is the sampling time.

Mean collection efficiencies for each collection device were calculated by dividing the measured cytokine concentrations collected by each sampling device (Equation 2) by the estimated airborne cytokine concentration (Equation 1) as summarized below in Equation 3:
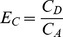
(3)Where *E_C_* is the collection efficiency of the device, *C_D_* is the measured concentration of cytokine collected by the sampling device, and *C_A_* is the estimated airborne cytokine concentration calculated as described in Equation 1.

Mean collection efficiencies and 95% CI values for all six cytokines and mean collection efficiencies for individual cytokines using each device were calculated.

### Experiment 2: Cytokine Stability, Use of Filter Centrifugation Concentration and Effect of Sampling Time and Cytokine Concentrations on Assay Repeatability

Potential degradation of cytokines during nebulization was evaluated (Figure 7) by calculating the total amount of cytokine present in the nebulization solution at 10 minute intervals from 0 to 30 minutes. For this experiment, a HEART nebulizer generated aerosols of a solution of six human cytokines containing 25 pg/ml of each cytokine in 1X PBS buffer prepared as described in Experiment 1. Samples were removed from the nebulizer solution at the time points listed above using a syringe and then assayed by multiplex immunoassay.

Preliminary experiments indicated approximately 50% sample loss due to use of the rotary centrifugal evaporator for concentration of filter extracts. Thus, use of Amicon Ultra-4 3000 MWCO centrifugal filter concentrators (Millipore, Billerica, MA) was evaluated for 10-fold concentration of solutions containing human cytokines (Figure 7). For this experiment, cytokine recovery from solution was determined by spike-recovery experiments using human cytokine standards at a concentration of 10 pg/mL in 1X PBS buffer prepared as described in Experiment 1. Passivation or pre-treatment of the centrifugal filter concentrators with a solution of 1% powdered milk in 1X PBS, as suggested by the manufacturer, was also performed for comparison to untreated filters to assess whether passivation yielded improved recovery of cytokines. Additionally, the mean collection efficiency of filter samples generated in Experiment 2, extracted and then concentrated using centrifugal filter concentrators was calculated as described in Experiment 1 (Equations 1–3) and compared to results obtained from filter extracts concentrated using rotary centrifugal evaporation in Experiment 1.

Results from Experiment 1 indicated collection of aerosolized cytokines in the pg/m^3^ range which when assayed by multiplex immunoassay included reported levels near or slightly above the manufacturer’s reported minimum detectable concentrations (MinDC) which range from 0.01 pg/mL to 0.48 pg/mL and vary by cytokine. According to the assay manufacturer, MinDC is calculated using StatLIA® Immunoassay Analysis Software (Brendan Technologies Inc., Carlsbad, CA) by mathematically determining what the empirical value for MinDC would be if an infinite number of standard concentrations were run in an assay under identical conditions. Min DC values for IL-1 beta, IL-7, IL-8, IL-13, IFN-gamma, and TNF-alpha were 0.06, 0.12, 0.11, 0.48, 0.29, and 0.05 pg/mL, respectively.

To evaluate repeatability of assay measurements at the manufacturer’s MinDC range for the multiplex immunoassay as well as to investigate the effect of increasing sampling times of aerosols containing cytokines present in the pg/m^3^ range (Figure 7), we prepared solutions of six human cytokine standards as described in Experiment 1, at three different concentrations: 25 pg/mL; 10 pg/mL; and 1 pg/mL. Sampling times and starting concentrations were selected based on the goal of achieving collected cytokine amounts below, near, and one to two orders of magnitude above the manufacturer’s reported MinDC of the multiplex immunoassay.

Assumptions regarding sample loss and recovery were made based on earlier experiments and used in calculations to determine optimal cytokine concentrations and sampling times to produce the desired range of cytokine concentrations in the concentrated filter extracts.

After a chamber stabilization period of 15 minutes, solutions containing 25 pg/mL, 10 pg/mL, or 1 pg/mL were nebulized and delivered into a single-pass aerosol chamber and collected by Teflo™ Teflon® filter sampling cassettes. Duplicate filter samples were collected for each concentration of cytokine standards at each of three sampling times: 10; 20; and 30 minutes. Additionally, duplicate filter samples of nebulized 1X PBS/1% BSA/0.01% Tween-20 solution containing no cytokine standards were collected at 10, 20 and 30 minutes to serve as negative control samples. Two separate trials were performed for each concentration and sampling time. One laboratory blank sample which received no air exposure was collected for each of the two trials. Volume of total nebulizer solution was recorded and aliquots of nebulizer solution were taken at 0 minutes and 30 minutes after the start of nebulization.

All filter washes were performed as described in Experiment 1. However, Amicon Ultra-4 3000 MWCO centrifugal filter concentrators (Millipore, Billerica, MA) were employed in this experiment for 10-fold concentration of all filter washes and 1 mL nebulizer solution aliquots.

All samples were assayed by multiplex immunoassay and analyzed to determine cytokine levels (pg/mL) present in each sample. Values for samples with cytokine levels below the lowest standard of the assay (0.03 pg/mL) were calculated using linear regression methods of the four lowest data points of the standard curve for each cytokine. First, mean and standard deviation values were calculated for the median fluorescent intensity values of two sample matrix blanks assayed in duplicate (4 sample matrix blank intensity values/cytokine). Next, a limit of detection (LOD) value in intensity units was calculated for each cytokine by calculating the sum of the mean intensity value of the sample blank plus 3 SDs of the mean. The four lowest standards of the assay that were equal to or greater than this calculated intensity-based LOD value were then selected for inclusion in the linear regression analysis. Estimations of each sample concentration (pg/mL) measuring below the lowest standard of the assay were then made by using the linear regression equation calculated for each cytokine to determine sample concentration (pg/mL) using sample median fluorescent intensity values as input values in the regression equation.

### Experiment 3: Effect of Storage Conditions

In order to evaluate handling and storage of human cytokine aerosols collected using filters, we compared storage of filters containing dried samples and concentrated filter extracts at two different temperatures to that of samples assayed immediately after collection (Figure 7). For this experiment, thirteen human cytokine standards (GM-CSF, IFN-gamma, IL-1 beta, IL-10, IL-12(p70), IL-13, IL-2, IL-4, IL-5, IL-6, IL-7, IL-8, and TNF-alpha, Millipore, Billerica, MA) were prepared in solution as described in Experiment 1 at a concentration of 50 pg/mL and added to the HEART nebulizer. The solution was nebulized into a single-pass aerosol chamber connected to Teflo™ Teflon® filter sampling cassettes. A total of six replicate filter samples were collected for each of five storage conditions utilizing a 20 minute sampling time:

1. No storage; wash, concentrate and assay immediately after collection.
2. Store at −20°C for 2 weeks, then wash, concentrate and assay.
3. Store at −80°C for 2 weeks, then wash, concentrate and assay.
4. Wash and concentrate. Store at −20°C for 2 weeks, then assay.
5. Wash and concentrate. Store at −80°C for 2 weeks, then assay.

A total of 30 filters were collected in the study (5 conditions, 6 replicate filters/condition) and assayed in duplicate for a total of 60 measurements. Location of each filter collected for each condition was randomized across all sample collection runs. Five filter blanks (one for each storage condition) were collected receiving airflow containing 1X PBS/1% BSA/0.01% Tween-20 buffer only and served as negative control samples. Additionally, five laboratory blank filters that received no airflow were collected.

All filter washes were performed as described previously and concentrated 10-fold using Amicon Ultra-4 3000 MWCO centrifugal filter concentrators. Values for samples displaying cytokine measurements below the lowest standard of the assay were calculated as described in Experiment 2.

### Statistical Analyses

A Kruskal-Wallis (non-parametric) analysis of variance test was used to determine if differences between sampling devices compared in Experiment 1 were significant. If the analysis of variance test indicated significant differences among the sampling devices and data were approximately Gaussian, student’s t-tests were performed to pairwise compare mean collection efficiencies of samplers.

In Experiment 2, Tukey box plots were generated to evaluate cytokine degradation due to nebulization and significant differences in cytokine levels over 30 minutes of nebulization were determined using a Kruskal-Wallis analysis of variance test. Mean cytokine amounts (pg, 95% CI) for all six cytokines for each sampling time and concentration were plotted and compared using a Kruskal- Wallis analysis of variance test to test for significant differences. Additionally, the variability of the cytokine measurements made below, near and one to two orders of magnitude above the MinDC using our method was assessed.

In Experiment 3, Kruskal-Wallis analysis of variance tests were performed to determine any cytokine specific effects of two different storage temperatures (−20°C vs. −80°C) on samples stored as filters and as concentrated filter extracts as compared to samples assayed immediately after collection. Mean cytokine levels (pg) for each storage condition were plotted using Tukey box-plots and compared using a Kruskal- Wallis analysis of variance test to test for significant differences among storage conditions globally across all thirteen cytokines. Student’s t-tests (approximately Gaussian data) were used to compare the four storage conditions to the control condition (no storage).

All statistical analyses were performed using GraphPad Prism version 5.00 for Windows, (GraphPad Software, San Diego, CA).

## Supporting Information

Figure S1 **Stability of cytokines during nebulization.** Tukey box plot of mean levels (pg/mL) for all six cytokines over 30 minutes of nebulization. Differences in mean cytokine amounts across sampling times up to 30 minutes are not significant (Kruskal-Wallis, P>0.05), (N = 12, 2 samples for each of 6 cytokines).
(TIF)

Figure S2 **Effect of sampling time and initial starting concentration on IFN-gamma collection.** Measured levels of IFN-gamma (pg) collected using Teflon filters in aerosol chamber for 10, 20 and 30 minutes at starting concentrations of 1, 10 and 25 pg/mL. 95% CI bars are shown (N = 8, 4 filters assayed in duplicate).
(TIF)

Figure S3 **Effect of sampling time and initial starting concentration on IL-1 beta collection.** Measured levels of IL-1 beta (pg) collected using Teflon filters in aerosol chamber for 10, 20 and 30 minutes at starting concentrations of 1, 10 and 25 pg/mL. 95% CI bars are shown (N = 8, 4 filters assayed in duplicate).
(TIF)

Figure S4 **Effect of sampling time and initial starting concentration on IL-13 collection.** Measured levels of IL-13 (pg) collected using Teflon filters in aerosol chamber for 10, 20 and 30 minutes at starting concentrations of 1, 10 and 25 pg/mL. 95% CI bars are shown (N = 8, 4 filters assayed in duplicate).
(TIF)

Figure S5 **Effect of sampling time and initial starting concentration on IL-7 collection.** Measured levels of IL-7 (pg) collected using Teflon filters in aerosol chamber for 10, 20 and 30 minutes at starting concentrations of 1, 10 and 25 pg/mL. 95% CI bars are shown (N = 8, 4 filters assayed in duplicate).
(TIF)

Figure S6 **Effect of sampling time and initial starting concentration on IL-8 collection.** Measured levels of IL-8 (pg) collected using Teflon filters in aerosol chamber for 10, 20 and 30 minutes at starting concentrations of 1, 10 and 25 pg/mL. 95% CI bars are shown (N = 8, 4 filters assayed in duplicate).
(TIF)

Figure S7 **Effect of sampling time and initial starting concentration on TNF-alpha collection.** Measured levels of TNF-alpha (pg) collected using Teflon filters in aerosol chamber for 10, 20 and 30 minutes at starting concentrations of 1, 10 and 25 pg/mL. 95% CI bars are shown (N = 8, 4 filters assayed in duplicate).
(TIF)

Figure S8 **Effect of storage condition on mean cytokine levels for GM-CSF.** Tukey box plots showing effect of five different storage conditions on recovered amounts (pg) of GM-CSF. (N = 12, 6 filters assayed in duplicate).
(TIF)

Figure S9 **Effect of storage condition on mean cytokine levels for IFN-gamma.** Tukey box plots showing effect of five different storage conditions on recovered amounts (pg) of IFN-gamma. (N = 12, 6 filters assayed in duplicate).
(TIF)

Figure S10 **Effect of storage condition on mean cytokine levels for IL-1 beta.** Tukey box plots showing effect of five different storage conditions on recovered amounts (pg) of IL-1 beta. (N = 12, 6 filters assayed in duplicate).
(TIF)

Figure S11 **Effect of storage condition on mean cytokine levels for IL-10.** Tukey box plots showing effect of five different storage conditions on recovered amounts (pg) of IL-10. (N = 12, 6 filters assayed in duplicate).
(TIF)

Figure S12 **Effect of storage condition on mean cytokine levels for IL-12p70.** Tukey box plots showing effect of five different storage conditions on recovered amounts (pg) of IL-12p70. (N = 12, 6 filters assayed in duplicate).
(TIF)

Figure S13 **Effect of storage condition on mean cytokine levels for IL-13.** Tukey box plots showing effect of five different storage conditions on recovered amounts (pg) of IL-13. (N = 12, 6 filters assayed in duplicate).
(TIF)

Figure S14 **Effect of storage condition on mean cytokine levels for IL-2.** Tukey box plots showing effect of five different storage conditions on recovered amounts (pg) of IL-2. (N = 12, 6 filters assayed in duplicate).
(TIF)

Figure S15 **Effect of storage condition on mean cytokine levels for IL-4.** Tukey box plots showing effect of five different storage conditions on recovered amounts (pg) of IL-4. (N = 12, 6 filters assayed in duplicate).
(TIF)

Figure S16 **Effect of storage condition on mean cytokine levels for IL-5.** Tukey box plots showing effect of five different storage conditions on recovered amounts (pg) of IL-5. (N = 12, 6 filters assayed in duplicate).
(TIF)

Figure S17 **Effect of storage condition on mean cytokine levels for IL-6.** Tukey box plots showing effect of five different storage conditions on recovered amounts (pg) of IL-6. (N = 12, 6 filters assayed in duplicate).
(TIF)

Figure S18 **Effect of storage condition on mean cytokine levels for IL-7.** Tukey box plots showing effect of five different storage conditions on recovered amounts (pg) of IL-7. (N = 12, 6 filters assayed in duplicate).
(TIF)

Figure S19 **Effect of storage condition on mean cytokine levels for IL-8.** Tukey box plots showing effect of five different storage conditions on recovered amounts (pg) of IL-8. (N = 12, 6 filters assayed in duplicate).
(TIF)

Figure S20 **Effect of storage condition on mean cytokine levels for TNF-alpha.** Tukey box plots showing effect of five different storage conditions on recovered amounts (pg) of TNF-alpha. (N = 12, 6 filters assayed in duplicate).
(TIF)

Figure S21 **HEART nebulizer output rate.** Output rate of HEART Nebulizer over 30 minutes at 10 psi with 100 mL starting volume of 1X Phosphate Buffered Saline (PBS) solution containing 1% Bovine Serum Albumin (BSA) and 0.01% Tween-20.
(TIF)
