# Supplementary figures and images for: Collection of Aerosolized Human Cytokines Using Teflon® Filters
Source: PLoS One. 2012 May 4;7(5):e35814. doi: 10.1371/journal.pone.0035814 (PMC3344827; doi:10.1371/journal.pone.0035814)

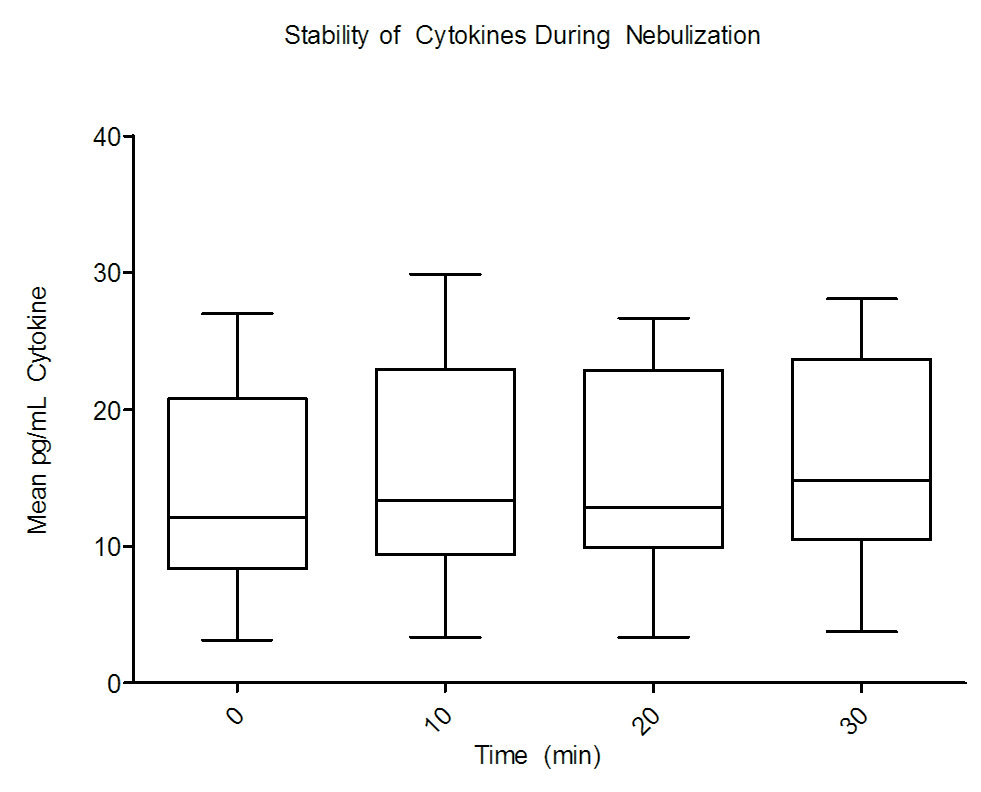

Supplement: Figure S1 — Stability of cytokines during nebulization. Tukey box plot of mean levels (pg/mL) for all six cytokines over 30 minutes of nebulization. Differences in mean cytokine amounts across sampling times up to 30 minutes are not significant (Kruskal-Wallis, P>0.05), (N = 12, 2 samples for each of 6 cytokines). (TIF) [file pone.0035814.s001.tif]

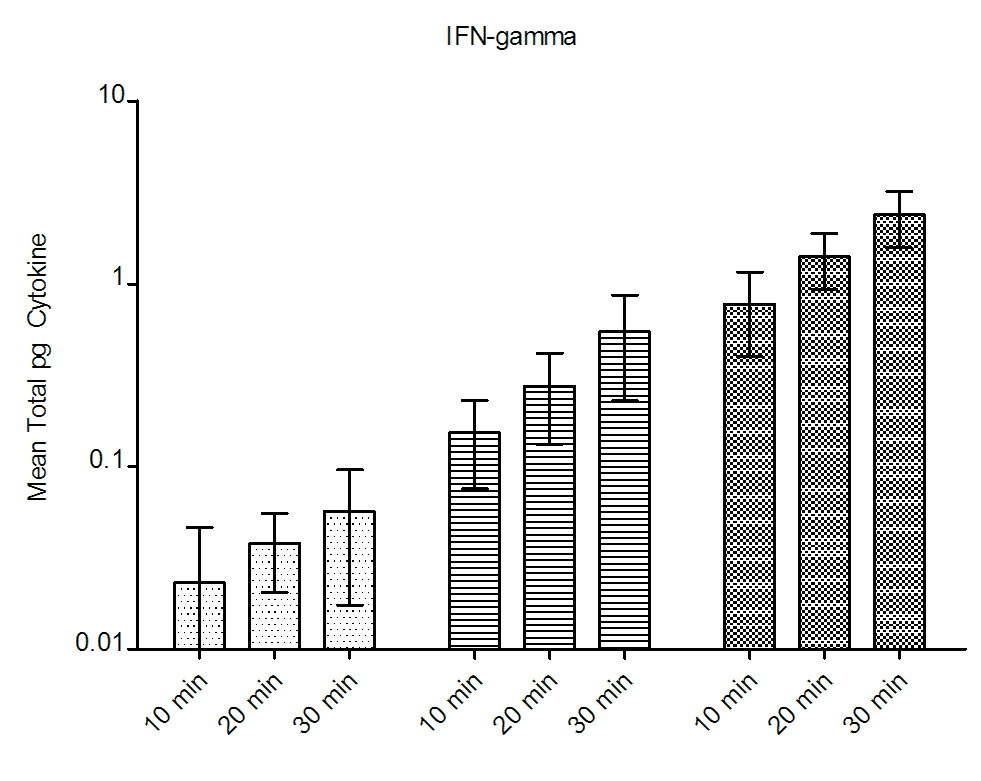

Supplement: Figure S2 — Effect of sampling time and initial starting concentration on IFN-gamma collection. Measured levels of IFN-gamma (pg) collected using Teflon filters in aerosol chamber for 10, 20 and 30 minutes at starting concentrations of 1, 10 and 25 pg/mL. 95% CI bars are shown (N = 8, 4 filters assayed in duplicate). (TIF) [file pone.0035814.s002.tif]

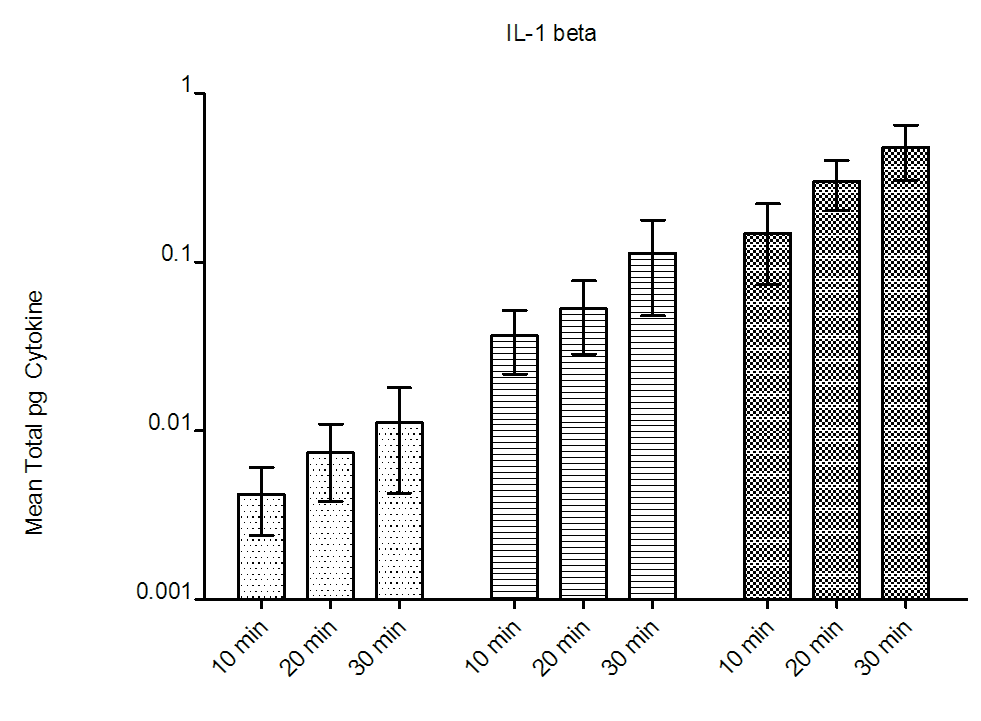

Supplement: Figure S3 — Effect of sampling time and initial starting concentration on IL-1 beta collection. Measured levels of IL-1 beta (pg) collected using Teflon filters in aerosol chamber for 10, 20 and 30 minutes at starting concentrations of 1, 10 and 25 pg/mL. 95% CI bars are shown (N = 8, 4 filters assayed in duplicate). (TIF) [file pone.0035814.s003.tif]

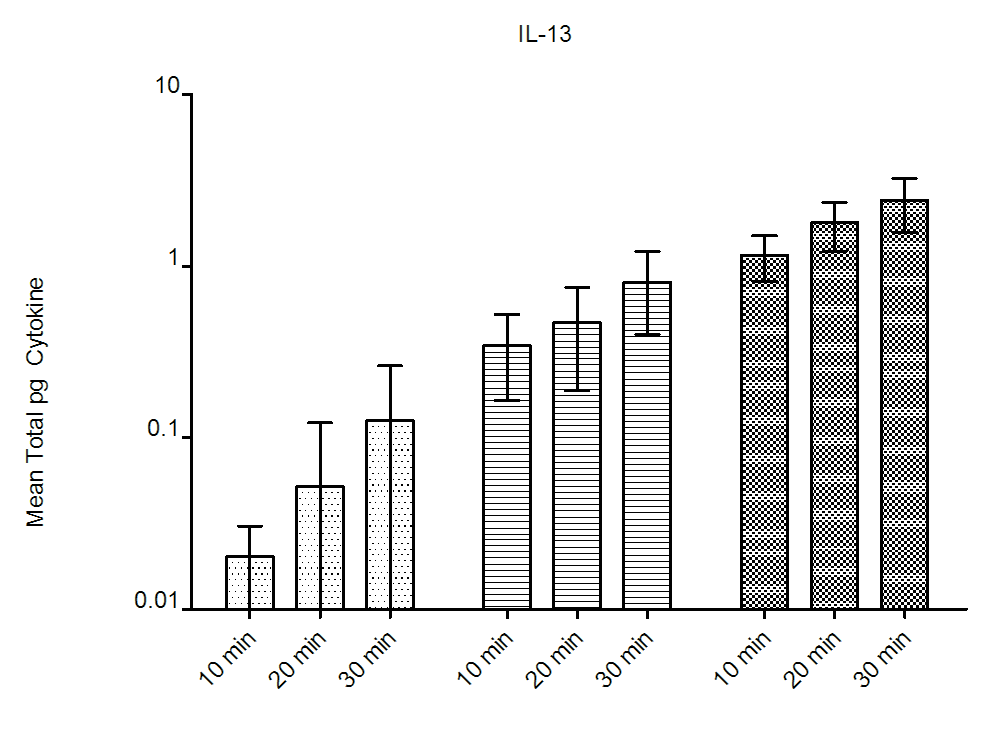

Supplement: Figure S4 — Effect of sampling time and initial starting concentration on IL-13 collection. Measured levels of IL-13 (pg) collected using Teflon filters in aerosol chamber for 10, 20 and 30 minutes at starting concentrations of 1, 10 and 25 pg/mL. 95% CI bars are shown (N = 8, 4 filters assayed in duplicate). (TIF) [file pone.0035814.s004.tif]

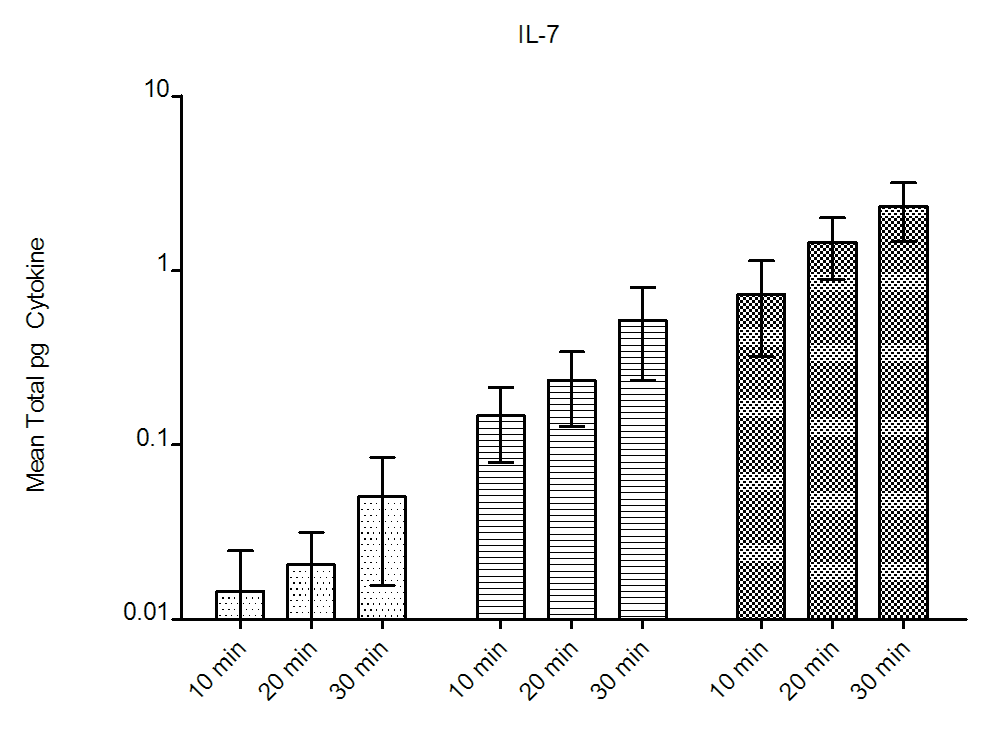

Supplement: Figure S5 — Effect of sampling time and initial starting concentration on IL-7 collection. Measured levels of IL-7 (pg) collected using Teflon filters in aerosol chamber for 10, 20 and 30 minutes at starting concentrations of 1, 10 and 25 pg/mL. 95% CI bars are shown (N = 8, 4 filters assayed in duplicate). (TIF) [file pone.0035814.s005.tif]

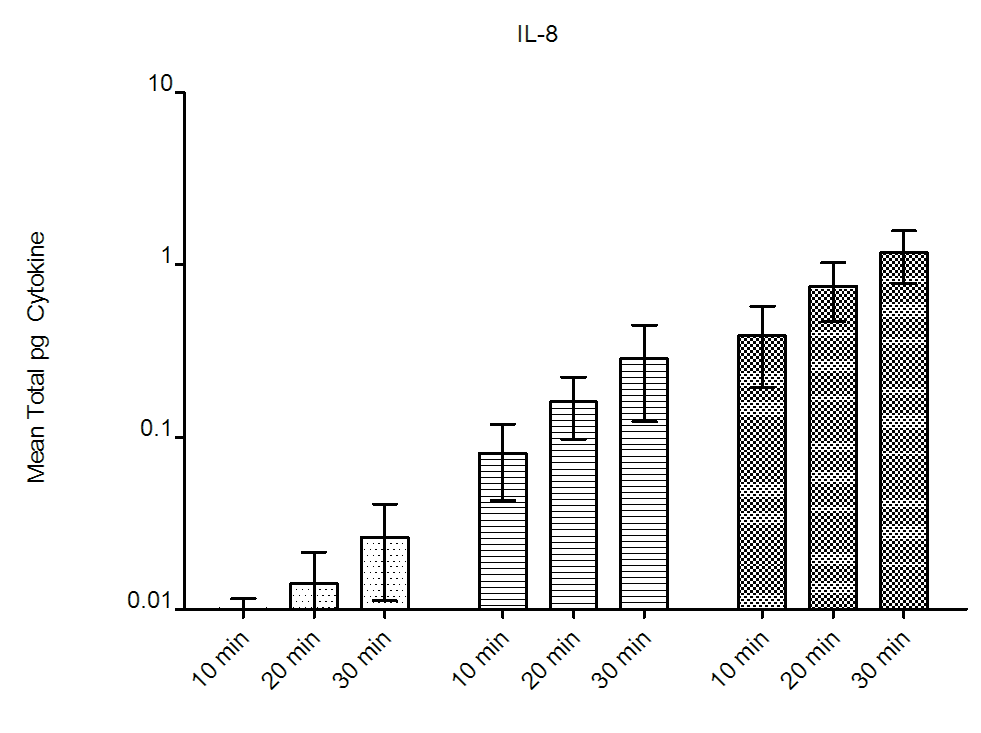

Supplement: Figure S6 — Effect of sampling time and initial starting concentration on IL-8 collection. Measured levels of IL-8 (pg) collected using Teflon filters in aerosol chamber for 10, 20 and 30 minutes at starting concentrations of 1, 10 and 25 pg/mL. 95% CI bars are shown (N = 8, 4 filters assayed in duplicate). (TIF) [file pone.0035814.s006.tif]

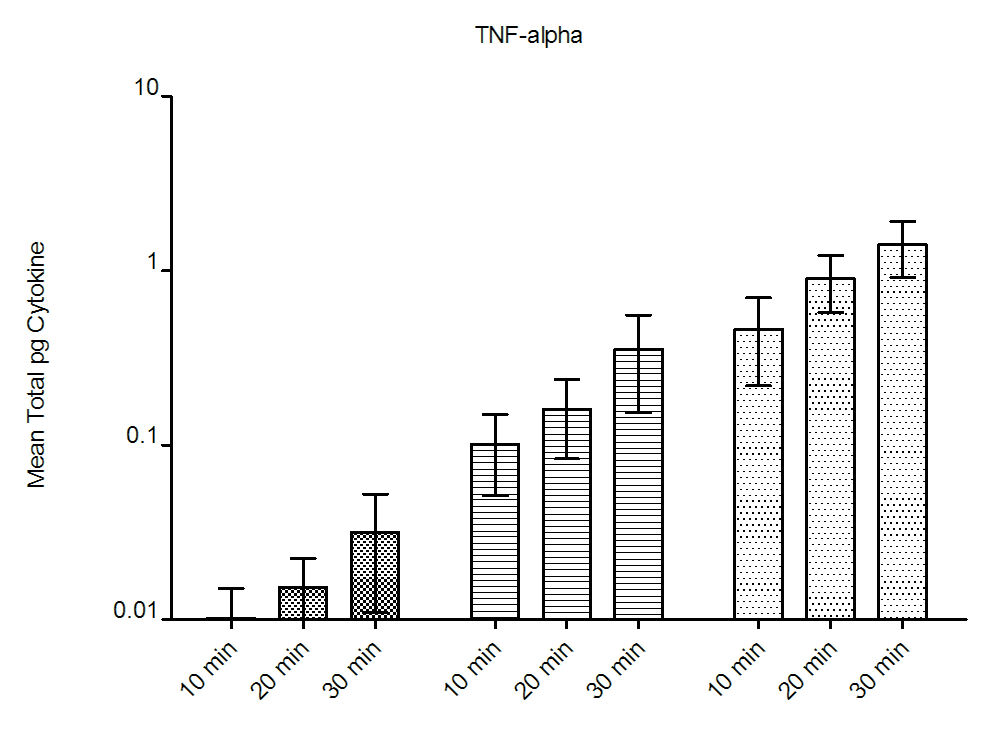

Supplement: Figure S7 — Effect of sampling time and initial starting concentration on TNF-alpha collection. Measured levels of TNF-alpha (pg) collected using Teflon filters in aerosol chamber for 10, 20 and 30 minutes at starting concentrations of 1, 10 and 25 pg/mL. 95% CI bars are shown (N = 8, 4 filters assayed in duplicate). (TIF) [file pone.0035814.s007.tif]

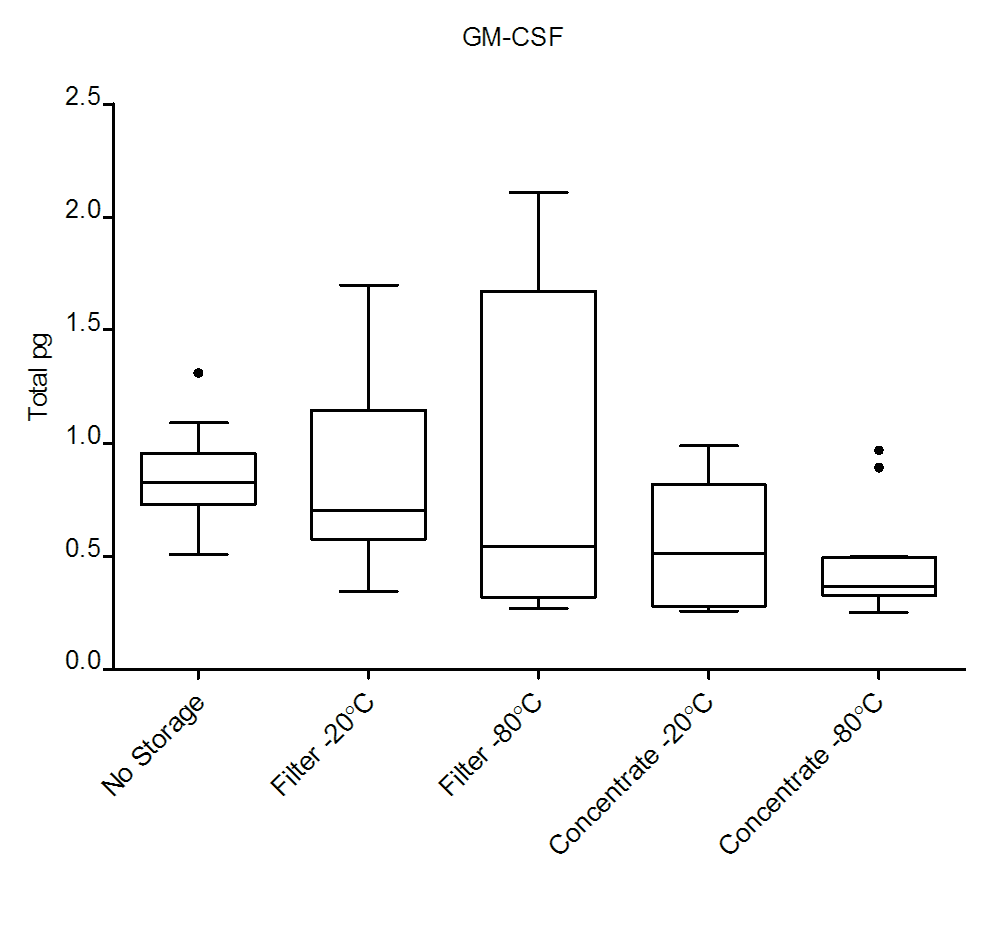

Supplement: Figure S8 — Effect of storage condition on mean cytokine levels for GM-CSF. Tukey box plots showing effect of five different storage conditions on recovered amounts (pg) of GM-CSF. (N = 12, 6 filters assayed in duplicate). (TIF) [file pone.0035814.s008.tif]

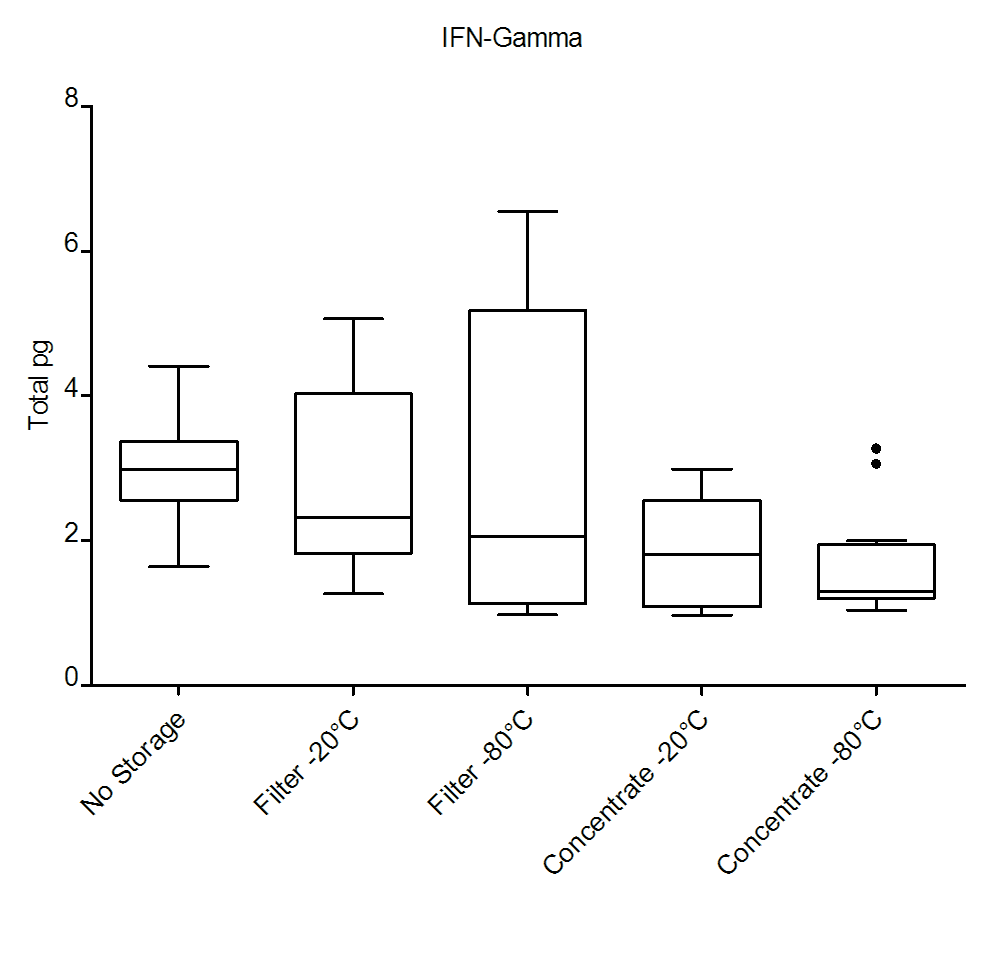

Supplement: Figure S9 — Effect of storage condition on mean cytokine levels for IFN-gamma. Tukey box plots showing effect of five different storage conditions on recovered amounts (pg) of IFN-gamma. (N = 12, 6 filters assayed in duplicate). (TIF) [file pone.0035814.s009.tif]

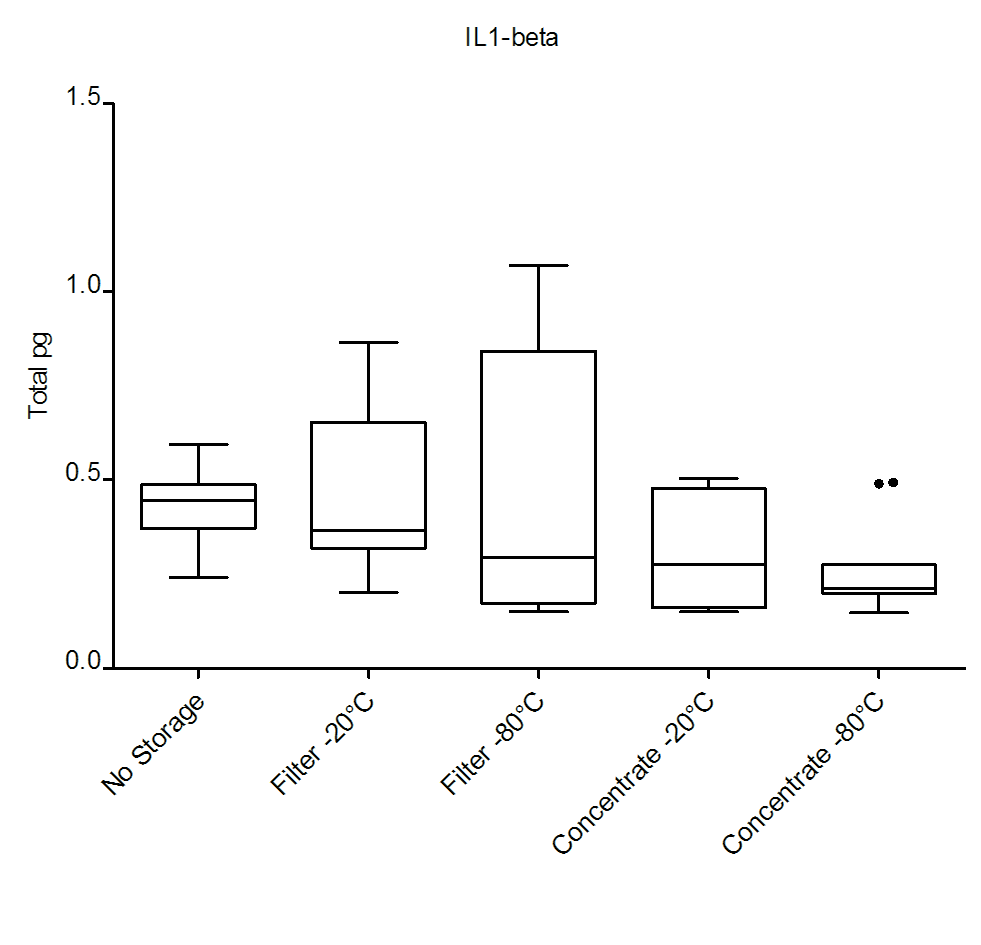

Supplement: Figure S10 — Effect of storage condition on mean cytokine levels for IL-1 beta. Tukey box plots showing effect of five different storage conditions on recovered amounts (pg) of IL-1 beta. (N = 12, 6 filters assayed in duplicate). (TIF) [file pone.0035814.s010.tif]

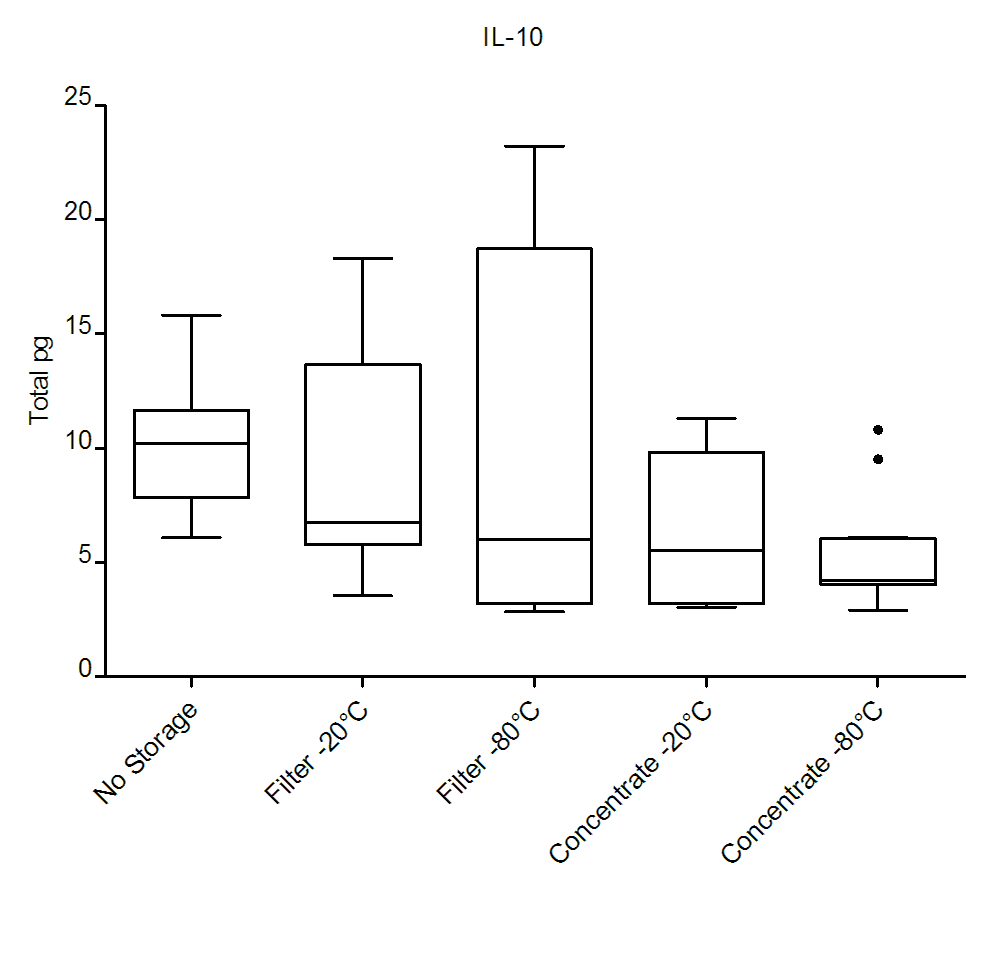

Supplement: Figure S11 — Effect of storage condition on mean cytokine levels for IL-10. Tukey box plots showing effect of five different storage conditions on recovered amounts (pg) of IL-10. (N = 12, 6 filters assayed in duplicate). (TIF) [file pone.0035814.s011.tif]

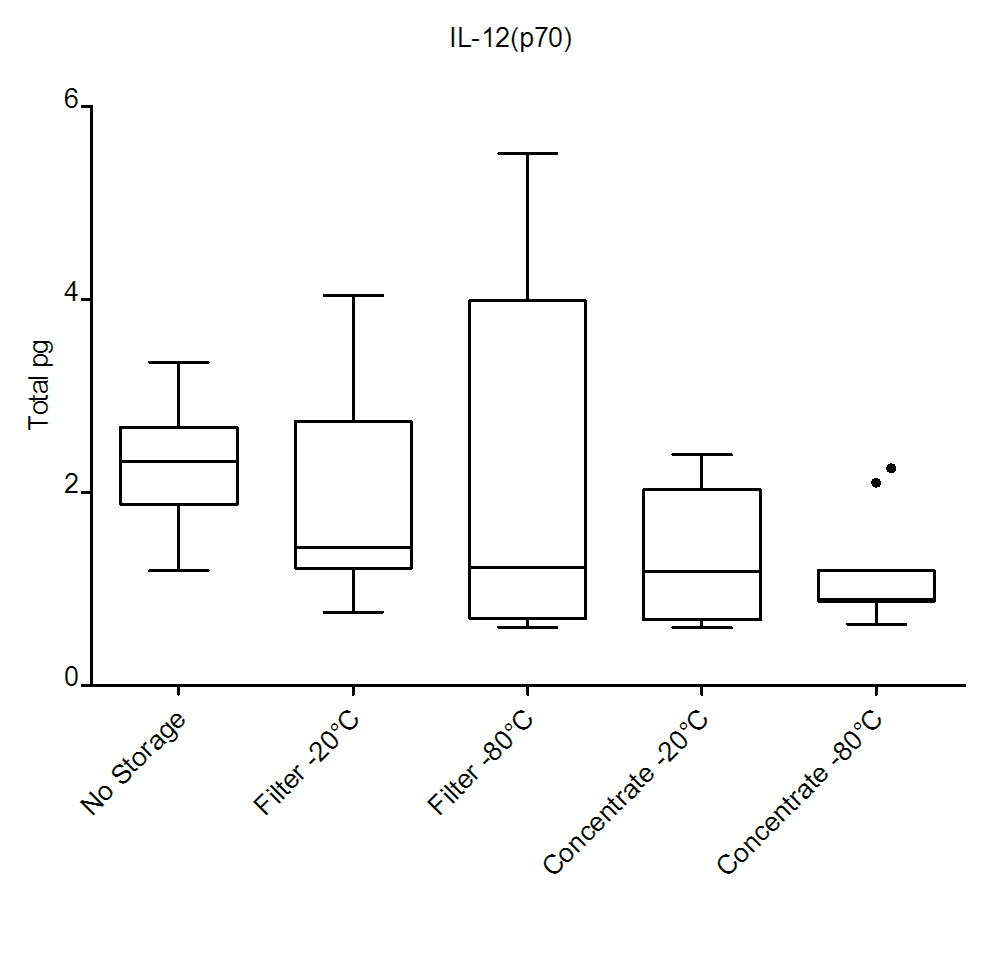

Supplement: Figure S12 — Effect of storage condition on mean cytokine levels for IL-12p70. Tukey box plots showing effect of five different storage conditions on recovered amounts (pg) of IL-12p70. (N = 12, 6 filters assayed in duplicate). (TIF) [file pone.0035814.s012.tif]

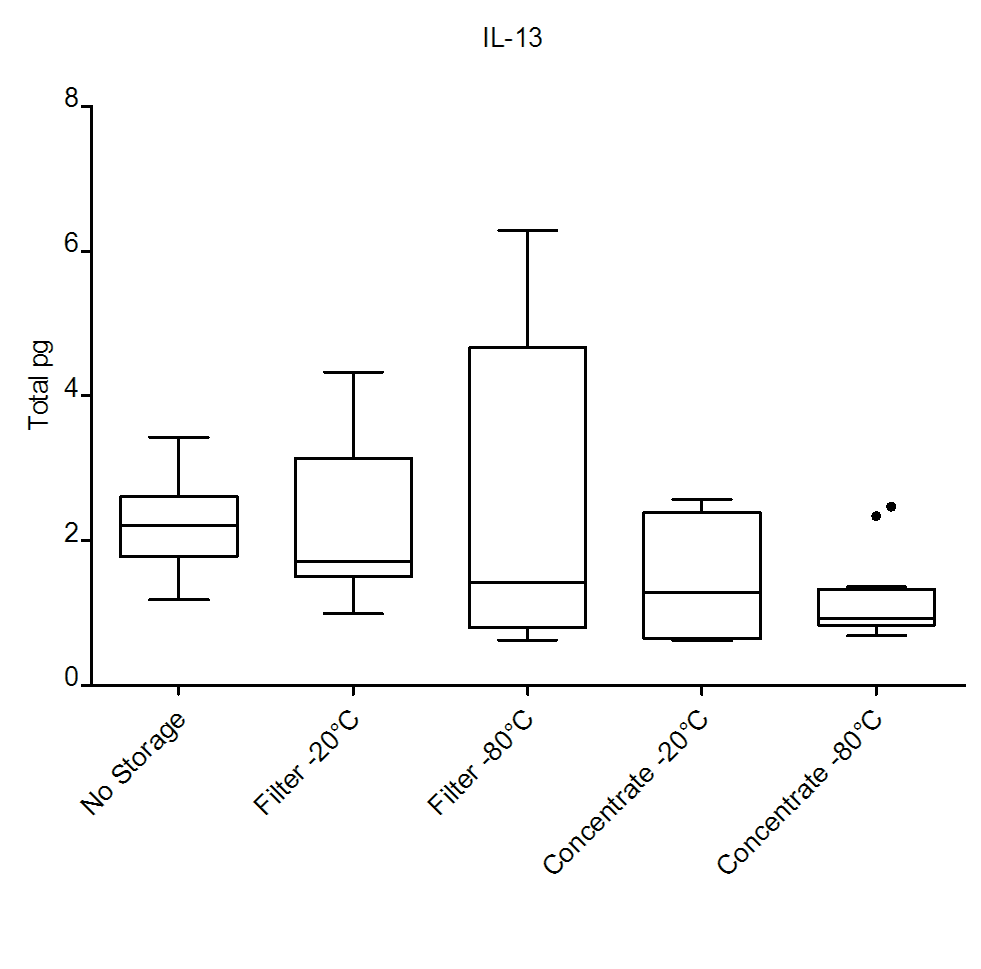

Supplement: Figure S13 — Effect of storage condition on mean cytokine levels for IL-13. Tukey box plots showing effect of five different storage conditions on recovered amounts (pg) of IL-13. (N = 12, 6 filters assayed in duplicate). (TIF) [file pone.0035814.s013.tif]

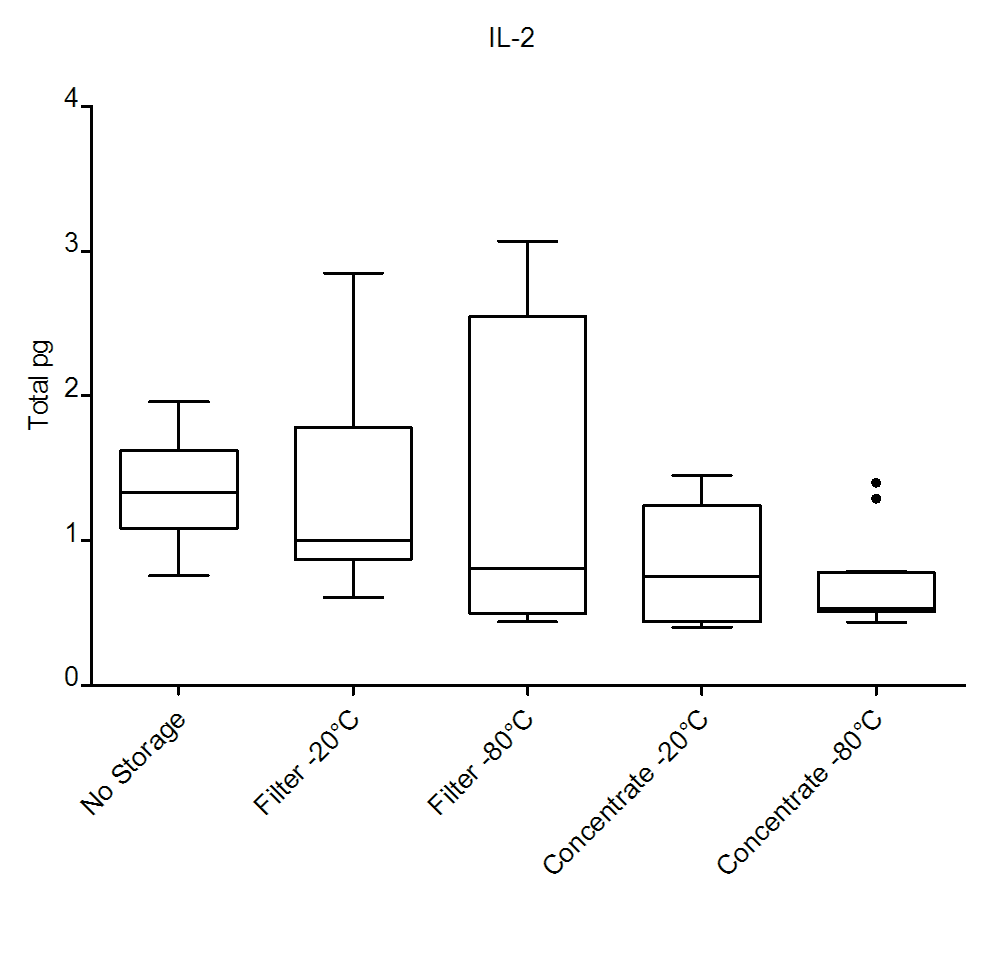

Supplement: Figure S14 — Effect of storage condition on mean cytokine levels for IL-2. Tukey box plots showing effect of five different storage conditions on recovered amounts (pg) of IL-2. (N = 12, 6 filters assayed in duplicate). (TIF) [file pone.0035814.s014.tif]

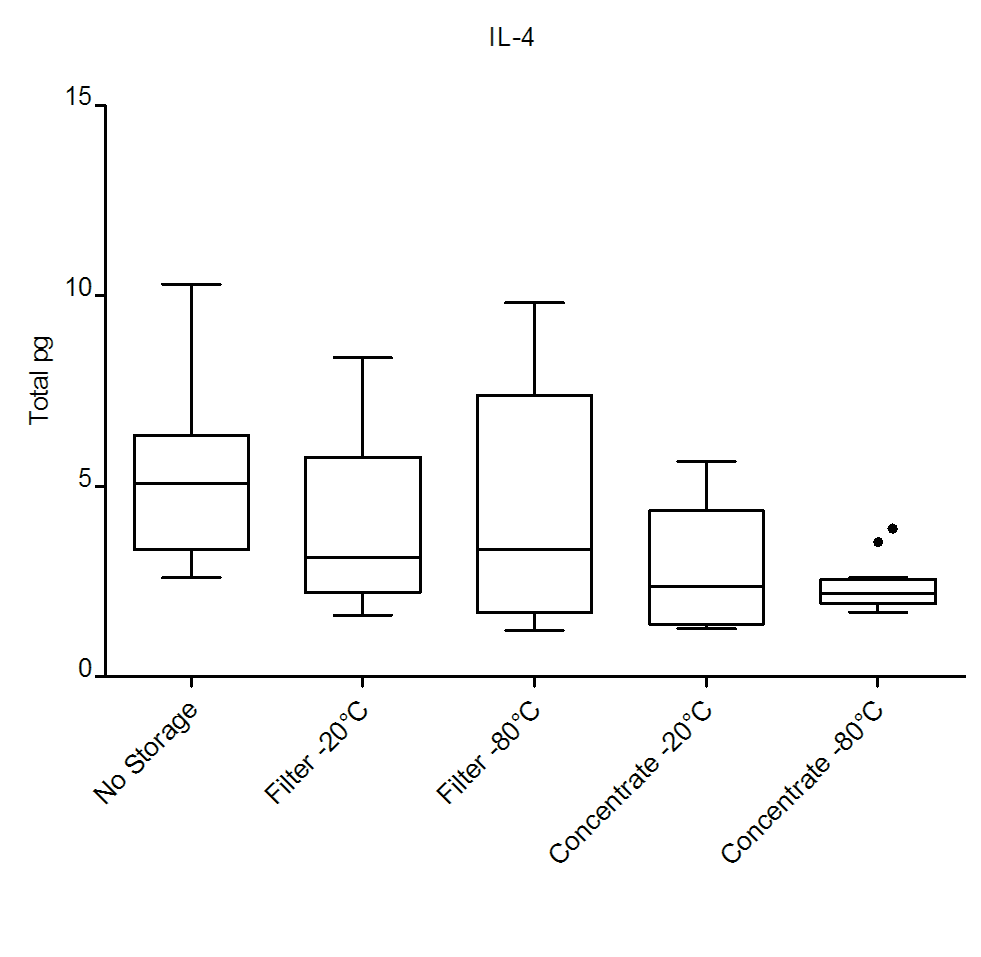

Supplement: Figure S15 — Effect of storage condition on mean cytokine levels for IL-4. Tukey box plots showing effect of five different storage conditions on recovered amounts (pg) of IL-4. (N = 12, 6 filters assayed in duplicate). (TIF) [file pone.0035814.s015.tif]

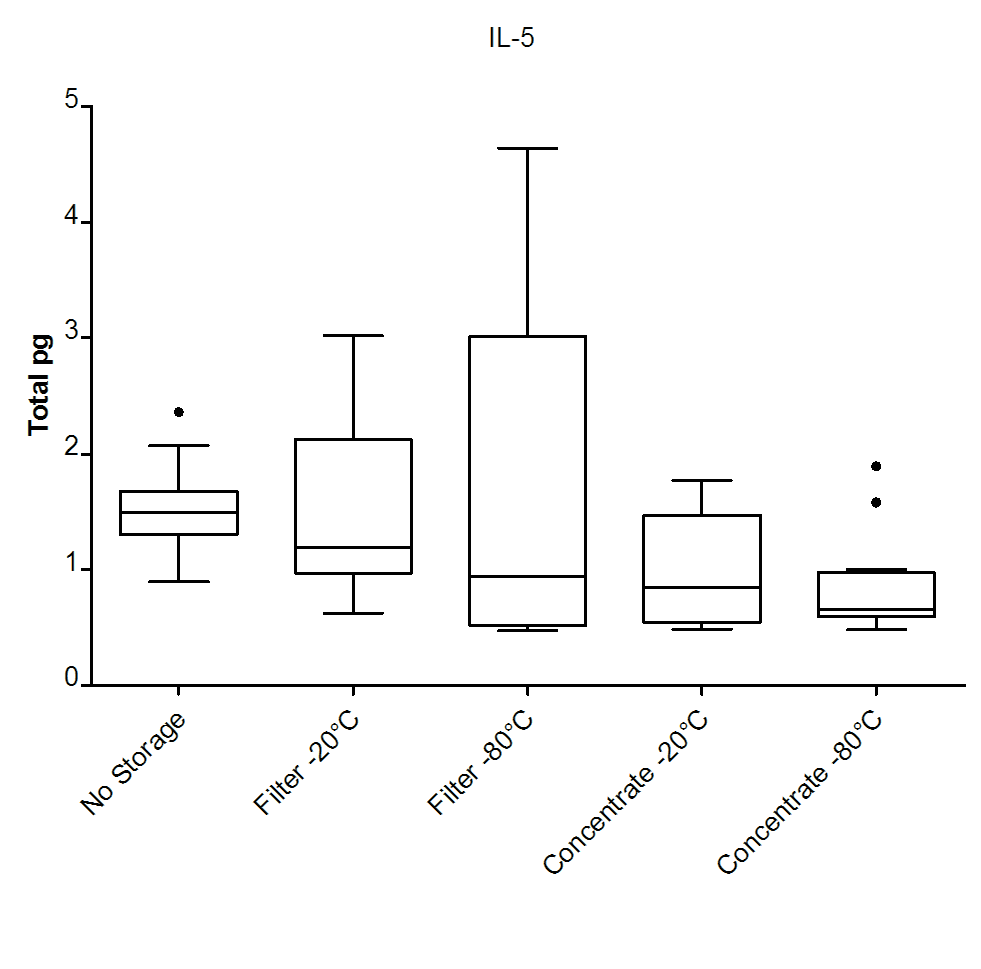

Supplement: Figure S16 — Effect of storage condition on mean cytokine levels for IL-5. Tukey box plots showing effect of five different storage conditions on recovered amounts (pg) of IL-5. (N = 12, 6 filters assayed in duplicate). (TIF) [file pone.0035814.s016.tif]

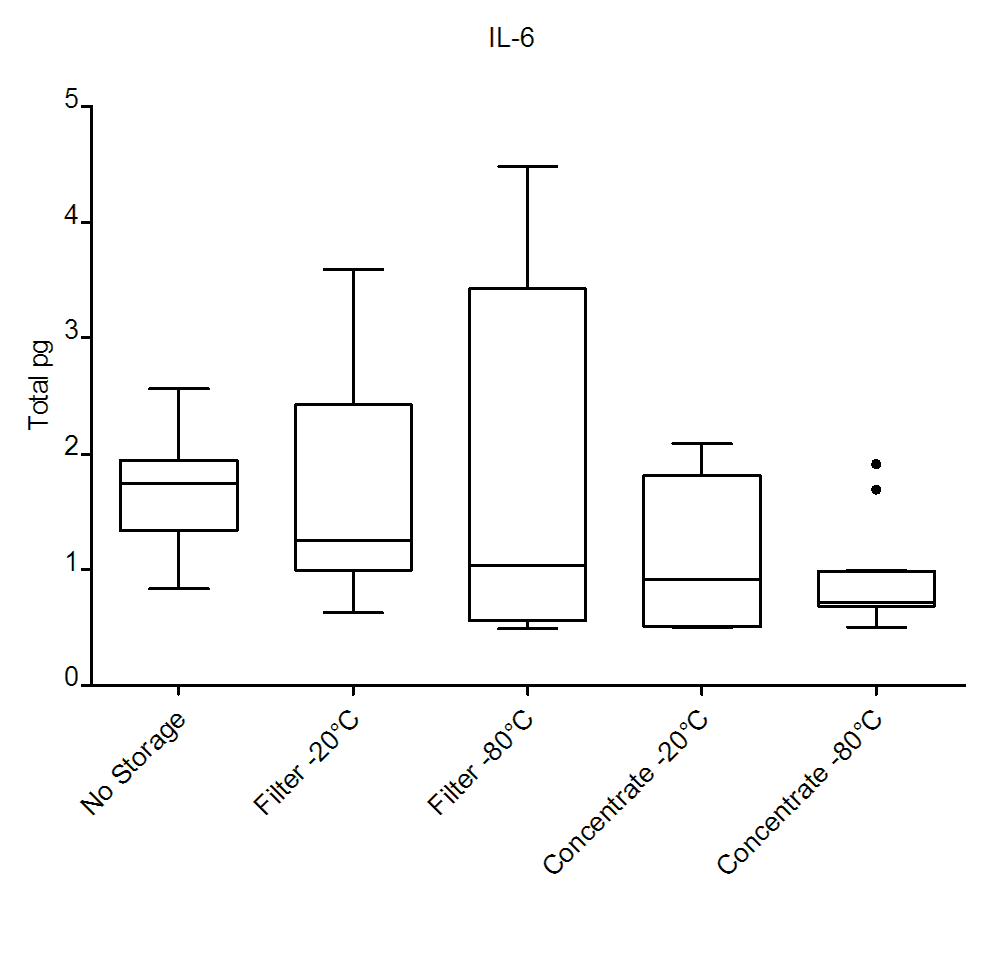

Supplement: Figure S17 — Effect of storage condition on mean cytokine levels for IL-6. Tukey box plots showing effect of five different storage conditions on recovered amounts (pg) of IL-6. (N = 12, 6 filters assayed in duplicate). (TIF) [file pone.0035814.s017.tif]

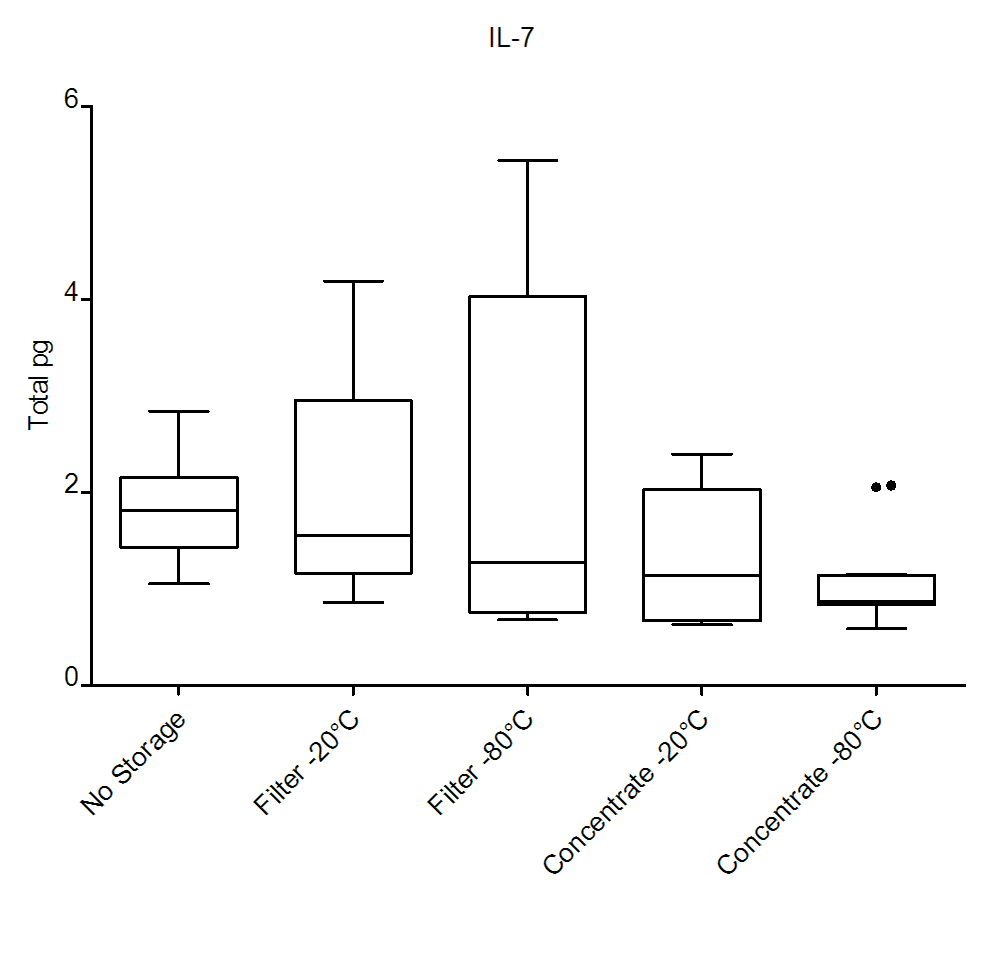

Supplement: Figure S18 — Effect of storage condition on mean cytokine levels for IL-7. Tukey box plots showing effect of five different storage conditions on recovered amounts (pg) of IL-7. (N = 12, 6 filters assayed in duplicate). (TIF) [file pone.0035814.s018.tif]

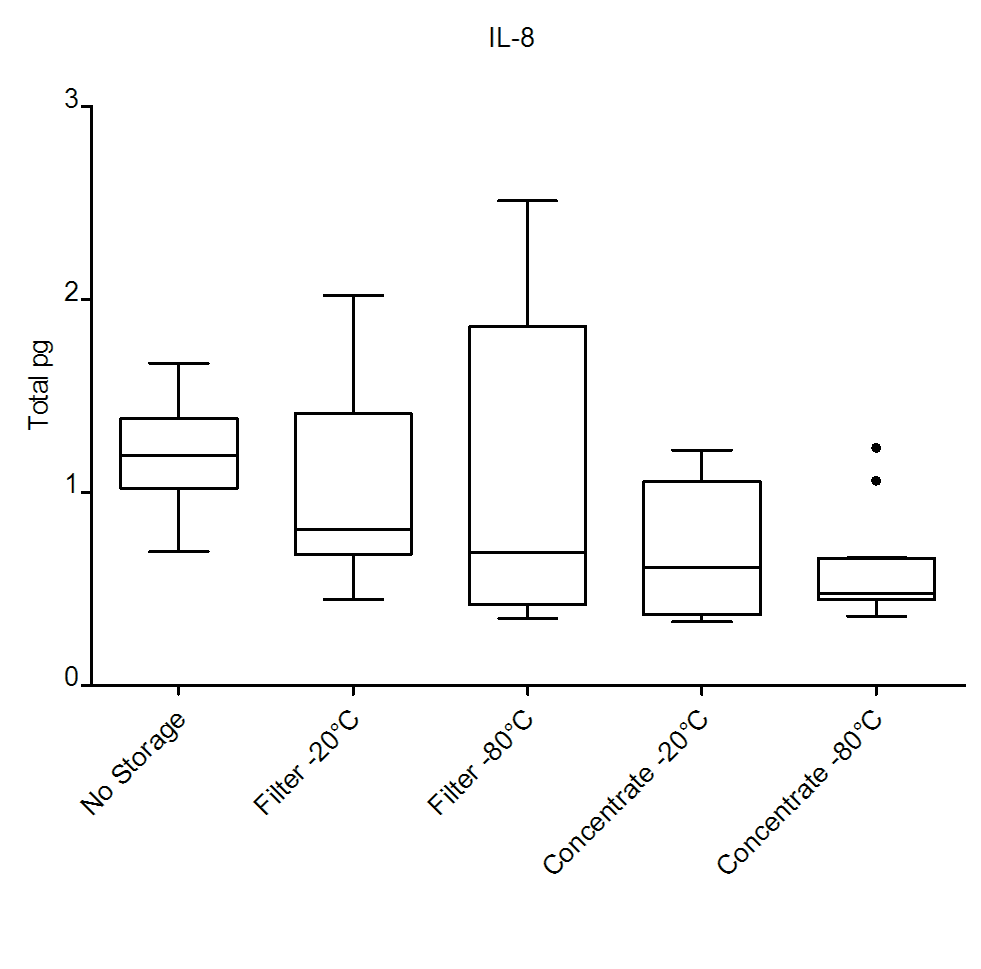

Supplement: Figure S19 — Effect of storage condition on mean cytokine levels for IL-8. Tukey box plots showing effect of five different storage conditions on recovered amounts (pg) of IL-8. (N = 12, 6 filters assayed in duplicate). (TIF) [file pone.0035814.s019.tif]

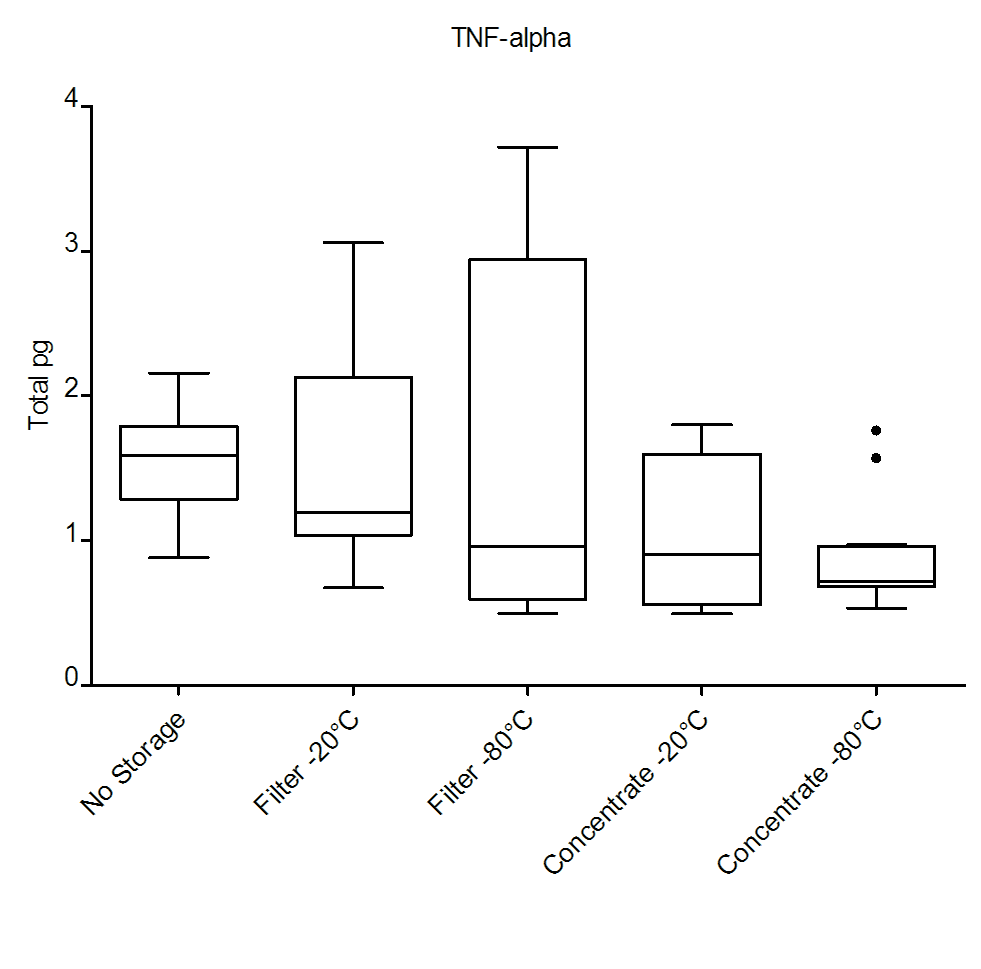

Supplement: Figure S20 — Effect of storage condition on mean cytokine levels for TNF-alpha. Tukey box plots showing effect of five different storage conditions on recovered amounts (pg) of TNF-alpha. (N = 12, 6 filters assayed in duplicate). (TIF) [file pone.0035814.s020.tif]

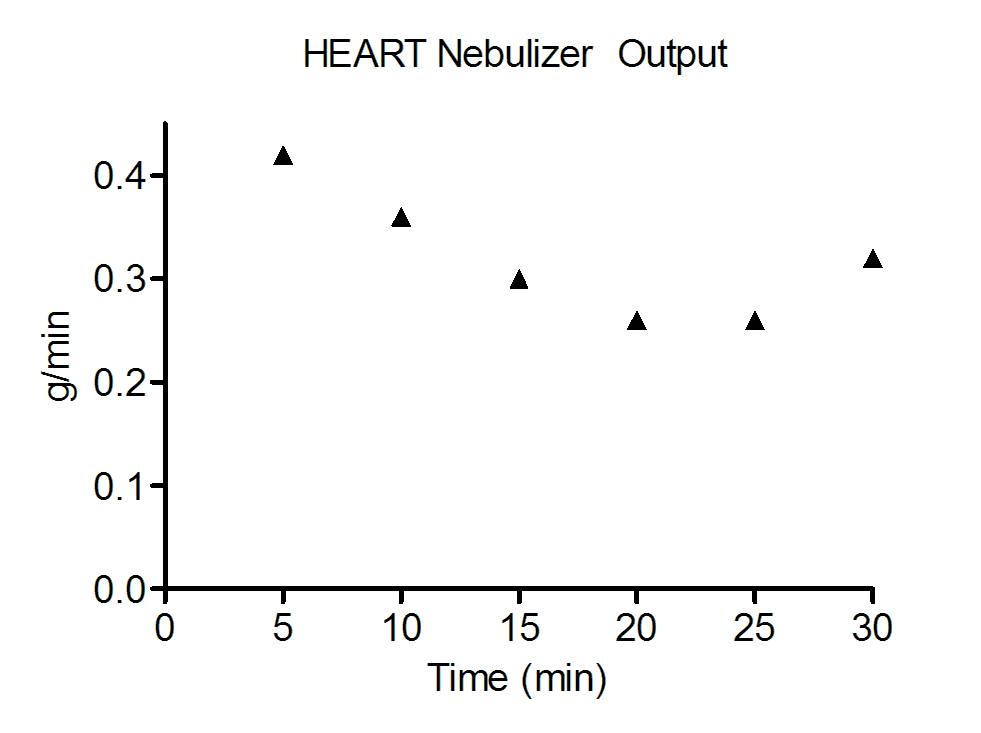

Supplement: Figure S21 — HEART nebulizer output rate. Output rate of HEART Nebulizer over 30 minutes at 10 psi with 100 mL starting volume of 1X Phosphate Buffered Saline (PBS) solution containing 1% Bovine Serum Albumin (BSA) and 0.01% Tween-20. (TIF) [file pone.0035814.s021.tif]
